# A portable non-invasive microwave based head imaging system using compact metamaterial loaded 3D unidirectional antenna for stroke detection

**DOI:** 10.1038/s41598-022-12860-8

**Published:** 2022-05-25

**Authors:** Mohammad Shahidul Islam, Mohammad Tariqul Islam, Ali F. Almutairi

**Affiliations:** 1 Department of Electrical, Electronic and Systems Engineering, Faculty of Engineering and Built Environment, Universiti Kebangsaan Malaysia, 43600 Bangi, Selangor Malaysia; 2 Electrical Engineering Department, College of Engineering and Petroleum, Kuwait University, 13060 Safat, Kuwait

**Keywords:** Electrical and electronic engineering, Biomedical engineering

## Abstract

A metamaterial (MTM) loaded compact three-dimensional antenna is presented for the portable, low-cost, non-invasive microwave head imaging system. The antenna has two slotted dipole elements with finite arrays of MTM unit cell and a folded parasitic patch that attains directional radiation patterns with 80% of fractional bandwidth. The operating frequency of the antenna is 1.95–4.5 GHz. The optimization of MTM unit cell is performed to increase the operational bandwidth, realized gain, and efficiency of the antenna within the frequency regime. It is also explored to improve radiation efficiency and gain when placed to head proximity. One-dimensional mathematical modelling is analyzed to precisely estimate the power distribution that validates the performance of the proposed antenna. To verify the imaging capability of the proposed system, an array of 9 antennas and a realistic three-dimensional tissue-emulating experimental semi-solid head phantom are fabricated and measured. The backscattered signal is collected from different antenna positions and processed by the updated Iterative Correction of Coherence Factor Delay-Multiply-and-Sum beamforming algorithm to reconstruct the hemorrhage images. The reconstructed images in simulation and experimental environment demonstrate the feasibility of the proposed system as a portable platform to successfully detect and locate the hemorrhages inside the brain.

## Introduction

Stroke is the 2nd most frequent cause of death and disability in developed countries. It is noteworthy that worldwide each year, approximately 16 million people are affected by stroke, of which 6 million die and another 6 million become disabled^1–3^. In the USA, about 796,000 people suffer a stroke, of which 650,000 are first strokes and 187,000 are recurrent strokes each year^4^. Stroke is characterized by acute onset neurological disability due to an underlying disruption of blood flow to the brain^4, 5^. Stroke is classified mainly into two categories: Ischemic stroke (IS) (~ 80%) and Hemorrhagic stroke (~ 20%)^6^. Magnetic resonance imaging (MRI) and computed tomography (CT) are the currently available medical diagnostic imaging modalities to detect a stroke. Although these existing technologies are highly sensitive, they are not easily accessible or affordable for patients in rural hospitals and can only be undertaken once the patient has been admitted to the hospital, apart from some centers where CT imaging has been made available through mobile ambulances. However, according to the World Health Organization (WHO), reliable and affordable medical imaging systems are not accessible to about three-quarters of the World’s population^7^. Furthermore, these existing imaging systems are very bulky^8^. Time to begin reperfusion is critical for limiting and perhaps reversing neurological disability. CT scans have drawbacks concerning exposure to radiation^9^, and cancer hazard^10^. MRI is more expensive and less widely available^11, 12^. Ultrasound imaging can be used to quantify intracranial and extracranial blood vessel anatomy and blood flow but requires expertise and is time-consuming^13^. PET imaging has been used to assess the ischemic penumbra but is not widely utilized^11, 12^. Hence, a portable, non-ionizing, low-cost, and highly accurate imaging system is urgently needed for the detection of stroke.

Microwave head imaging is nowadays an emerging technology for the medical diagnostic system that has drawn a significant interest among researchers all over the world because of its cost-effective, nonionizing, non-invasive, and portable features^13–18^. This technology is capable of detecting the early cancerous tissues, hemorrhages, and other internal changes in the human head from the variances in electrical properties of head^19, 20^. Radar scanning and tomography both are the approaches for image reconstruction, and in both cases, the antenna array is one of the main components as the performances of the antenna array determine the success of the system. Compactness and directionality are the major concerns in antenna geometry for microwave head imaging as compactness allows more elements within the available volume with portable and deployable features, and directionality ensures the enhancement of the radiated signal strengths by a consequent increase in the received scattered signals without backward signal interference^19, 21–23^. For satisfactory image resolution, wideband characteristics within the lower frequency are also required for efficient data acquisition. To get the compactness and wideband characteristics, a number of antennas have been designed by introducing the metamaterial (MTM) structure for the microwave imaging applications as MTMs are owing popularity because of their bandwidth enhancement, gain enhancement, and isolation capabilities^24–28^. It offers either negative permittivity or negative permeability or even both, by controlling electromagnetic waves, which makes it suitable to be used in any antenna by boosting the isolation among antenna elements. Several wideband antennas like MTM loaded antennas, patch antennas, flexible antennas, 3D antennas have been utilized in microwave head imaging system. However, each individual antenna has its own limitations in terms of size, radiation characteristics, the number of array elements, validation with head phantom, and specific absorption rate (SAR). A triangular patch antenna is presented in^29^ for microwave brain imaging. Two antennas are used in the imaging system to extract the scattering parameter. However, the image reconstruction capabilities remain limited due to the lower number of data point received from the two antennas array element. Besides, the homogeneous phantom is used to validate the performance that does not mimic the realistic head model. A semi-flexible monopole immersed brick antenna is presented in^30^ for microwave brain imaging system. The operating frequency of the antenna is 800 MHz to 1.2 GHz. Two prototypes are utilized with the head model to analyze the scattering performance of the antenna; however, there is no analysis on image reconstruction which limits the capability of the overall system. Moreover, the antenna dimension is not compact, and the resonance frequency below 1 GHz will tend to degrade the image resolution by showing blurry image although it improves the penetration. A flexible antenna presented in^31^ has been proposed for microwave on-body imaging system. The analysis reveals that the maximum SAR value of the presented antenna is 0.80 W/kg which is very high compared to other recent head imaging antennas^22, 32^. Moreover, there is no analysis on the microwave image reconstructions. In^33^, although the proposed flexible antenna has good image reconstruction capabilities, the head phantom verification is not suitable enough for realistic situations due to the homogeneous characteristics. The SAR analysis is also not reported in the article that limits the analysis of harmful effects due to the antenna radiation. Another clinical prototype is developed by EMTensor GmbH^13, 34^. The prototype consists of high relative permittivity-based 160 antenna arrays, which operates from 0.9 GHz to 1.8 GHz. This approach ensures better image resolution due to the large antenna setup. However, it suffers from low bandwidth and complex computing processes due to the involvement of a large number of antenna array. Moreover, due to the deficiency of dielectric properties in the coupling medium, the overall performance might be degraded with respect to time. A multi-frequency electromagnetic tomography (mfEMT) based laboratory prototype has been presented for detecting acute stroke in the human brain by applying frequency constrained sparse Bayesian learning algorithm^35^. The induction coils are used instead of antenna arrays for generating microwave signals. However, the brain tissues might be damaged due to variations of mutually high inductive reactance of the coil. The produced image resolution might also be degraded because of the excessive noise when a sinusoidal signal passes through the coil. Moreover, different methods have also been applied to the microwave stroke imaging technique. A newton-conjugate-gradient method has been presented in^36^ for the image reconstruction and development of microwave brain stroke imaging system. The method is developed in the Lp Banach space framework with nonconventional iterative solving scheme that smooth the effects associated with the reconstruction process. Although this method is effective in terms of regularized microwave imaging solutions, a low convergence speed has been identified in the image reconstruction and data acquisition process. The extension of the Lp spaces of conventional inversion method is also applied to some of the brain stroke imaging system analysis^37, 38^. However, the process is not straight forward in terms of generating the mathematical proof of convergence and the properties in image reconstruction technique. Furthermore, a variable exponent Lebesgue-space inversion technique has been presented to overcome the aforementioned limitations^39^. This algorithm is applied to the microwave imaging system for the quantitative analysis of the brain imaging inside the head. Although the proposed algorithm retrieves the reconstruction of brain stroke within the considered head model, the limitation still relies on the selection range of the exponent values that is an important consideration for the realistic 3D numerical model. Besides, to improve the quantitative time domain imaging algorithm, various approaches has been applied in microwave stroke imaging system. A hybrid time and frequency domain approach has been presented in^40^, where the method is combined of discontinuous Galerkin method and forward–backward time stepping algorithm. However, the limitation still presented in the resolution of the reconstructed images. Moreover, some other analysis has also been presented in terms of the time or frequency domain algorithm, but they have the weakness towards the computational cost and right frequency selection^41, 42^.

The antennas presented in^21, 43–45^ have used different multiple substrate layers to fabricate the prototype. Although the antenna design using multi dielectric layers have a significant effect on performance, the combination of superstrate must be chosen carefully since dielectric loss may adversely degrade the accuracy of the antenna gain and operating bandwidth. The harmful radiation effects of these antenna prototypes with SAR analysis are also not presented. Although the SAR analysis is presented in^22^, the antenna bandwidth decreases because of the effects of the superstrate layer. The inhomogeneous phantom is used to measure the scattering parameters rather than justifying the reconstructed images in microwave imaging system. In^44^, although the antenna shows high realized gain with small dimension, the analysis is only limited to identifying the scattering parameters. The analysis does not show any justification related to the experimental validation in microwave imaging system. An MTM based flexible wearable wideband antenna is presented at^46^ for the brain stroke diagnosis. The MTM unit cell increases the antenna bandwidth and enables unidirectional radiation, but the analysis of the radiation efficiency and gain when the antennas are placed with the head model is not presented. Another metasurface enhanced antenna is presented in^47^ for microwave brain imaging system. The antenna operates from 0.5 to 2.0 GHz and a tissue-mimicking gel phantom is used to analyze the imaging performance. The analysis concludes that inclusion of the metasurface elements can effectively enhance the return loss and improve the image reconstruction capabilities. Nevertheless, the issues rely on the tissue-mimicking gel phantom which does not properly emulate the real head tissue. Besides, the radiation effects of the metasurface antenna are not analyzed in the article. A metamaterial loaded monopole antenna is also presented in^48^ for the feasibility study of head imaging system. The analysis of metamaterial and its impact on the penetration signals are presented in this article. However, the liquid phantom is used to analyze the imaging target, which does not properly mimic the real tissue environment. In addition, RF coils in the head imaging system are well established system compared to microwave-based head imaging system. Basic concept of RF coils is the ‘antennae’ that broadcast the RF signal to the subject and receive the returned signal. Despite the extensive applications of the RF coil, few technical limitations still remain. The total acquisition time is generally more than eight (8) hours and inconvenient to achieve consistency for different live subjects^49^. Compared to the RF coil system; the microwave imaging system is real-time imaging system with portable features, where subjects can be easily positioned for diagnosis. Besides Maxwell’s law classified the potential RF coil effect as either non-thermal or thermal form. Non thermal effects are non-ionizing irradiation in RF range causes damage to unrelated tissue heating. By contrast, thermal effects may alter biological changes like thermoregulation, pain or burns since less than 2% of the transmitted RF power is absorbed by nuclei.

In this paper, an MTM loaded compact 3D antenna is presented for the portable microwave head imaging system that covers the frequency range of 1.95 to 4.5 GHz with 80% of fractional bandwidth. A finite array of MTM unit cell structures have been optimized to the antenna that has an impact on increasing the maximum efficiency from 66 to 89.6% and maximum realized gain from 4.55 to 6.01 dBi, respectively. Moreover, mathematical modelling is studied to estimate the antenna power distribution with the optimization of the metamaterial unit cell. A parametric analysis is performed to highlight the design parameters and examine the influences on the head tissue in terms of sensitivity. The E-field and H-field distribution with the effective medium theory is discussed to validate the antenna proximity performance. The radiation characteristics, efficiency, and gain are analyzed when the antenna is placed in proximity to the head model. The SAR distribution is also studied to show the antenna compatibility. The tissue emulating phantom and antenna prototypes are fabricated and measured to validate the overall imaging system performance. An updated Iterative Correction of Coherence Factor Delay-Multiply-and-Sum (IC-CF-DMAS) beamforming algorithm is utilized to reconstruct the hemorrhage images from the scattering parameters. The reconstructed images of the hemorrhages demonstrate the merits of the system as a portable platform and its potential in microwave head imaging systems.

## Design and analysis of metamaterial loaded 3D antenna

### Antenna geometry with metamaterial structure

**Figure 1:**
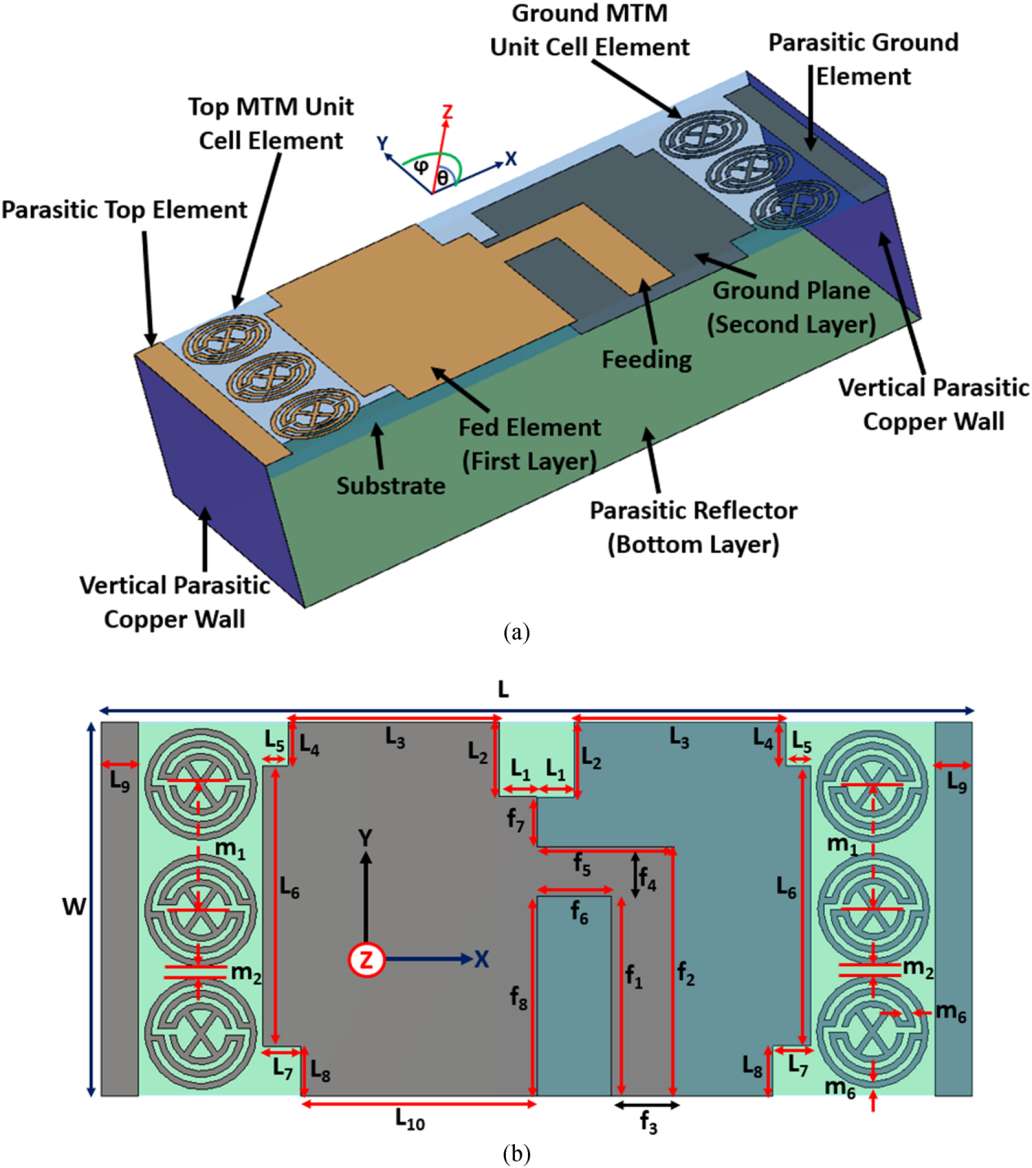
Schematic profile of the proposed MTM-loaded 3-D antenna. (**a**) Perspective view. (**b**) Top view. Dimensions (mm): L = 70, W = 30, L1 = 3, L2 = 6, L3 = 17, L4 = 3.5, L5 = 3, L6 = 22.5, L7 = 3, L8 = 4, L9 = 3, f1 = 16, f2 = 20, f3 = 5, f4 = 4, f5 = 11, f6 = 6, f7 = 4, f8 = 16, m1 = 10, m2 = 1, m6 = 0.5.

The proposed antenna schematics are depicted in Fig. 1. The design mechanism of the antenna starts with printing on the low loss Rogers RT5880 substrate slab where the permittivity, loss tangent and thickness are 2.2, 0.0009 and 1.57 mm, respectively. The antenna consists of a slotted dipole element with a finite array of MTM unit cell structure and a folded parasitic structure with two vertical and one bottom copper walls. 0.2 mm Copper plates are used for the vertical and bottom parasitic walls. The bottom layer parasitic wall acts as a parasitic reflector that has the same dimension as L × W. The fed and ground layer parasitic elements have a coupling effect with the antenna fed and ground, which drives current onto the parasitic walls that ensure the lower operating frequency.

**Figure 2:**
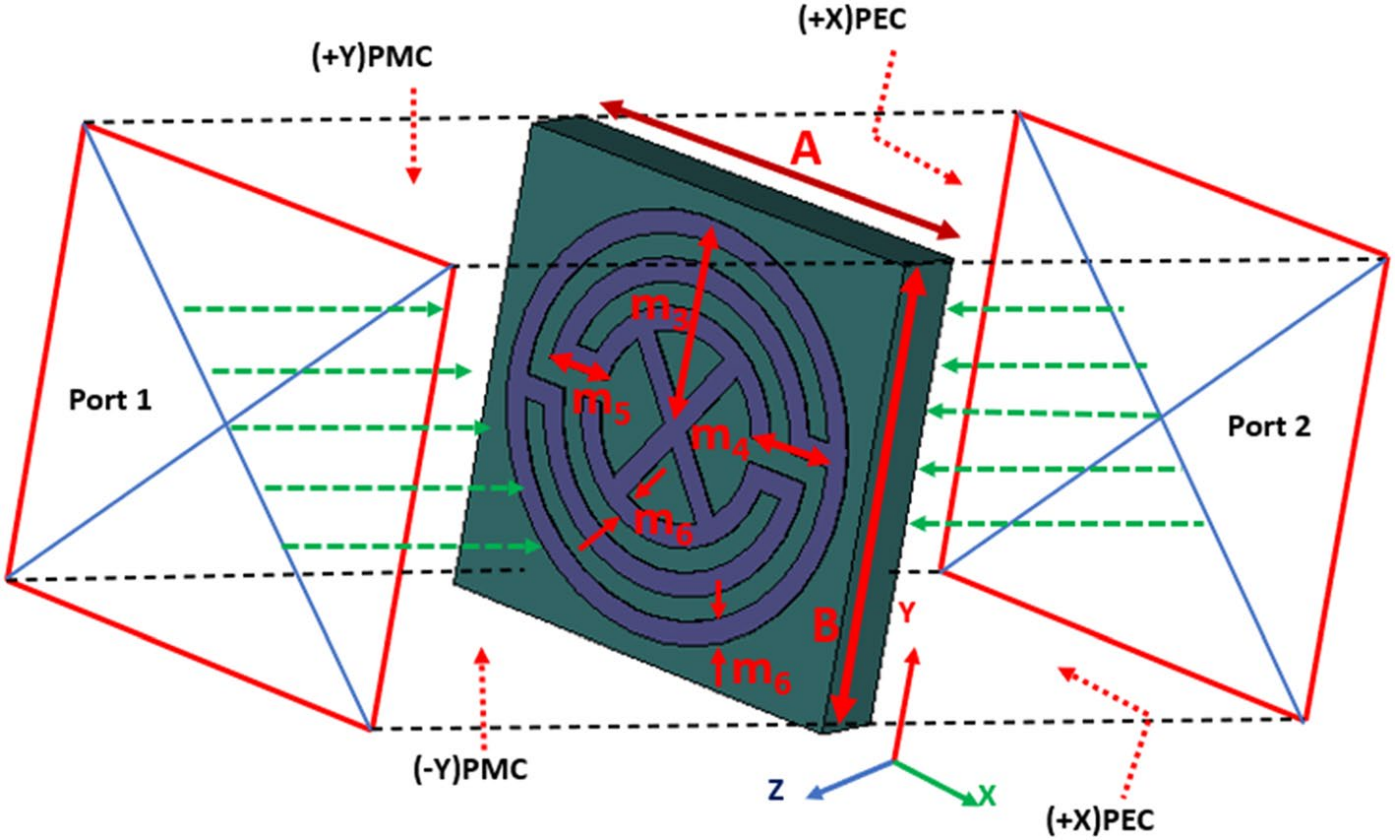
Simulation setup of MTM unit cell structure. Dimensions (mm): A = 10, B = 10, m3 = 4.5, m4 = 2, m5 = 1.5, and m6 = 0.5.

**Figure 3:**
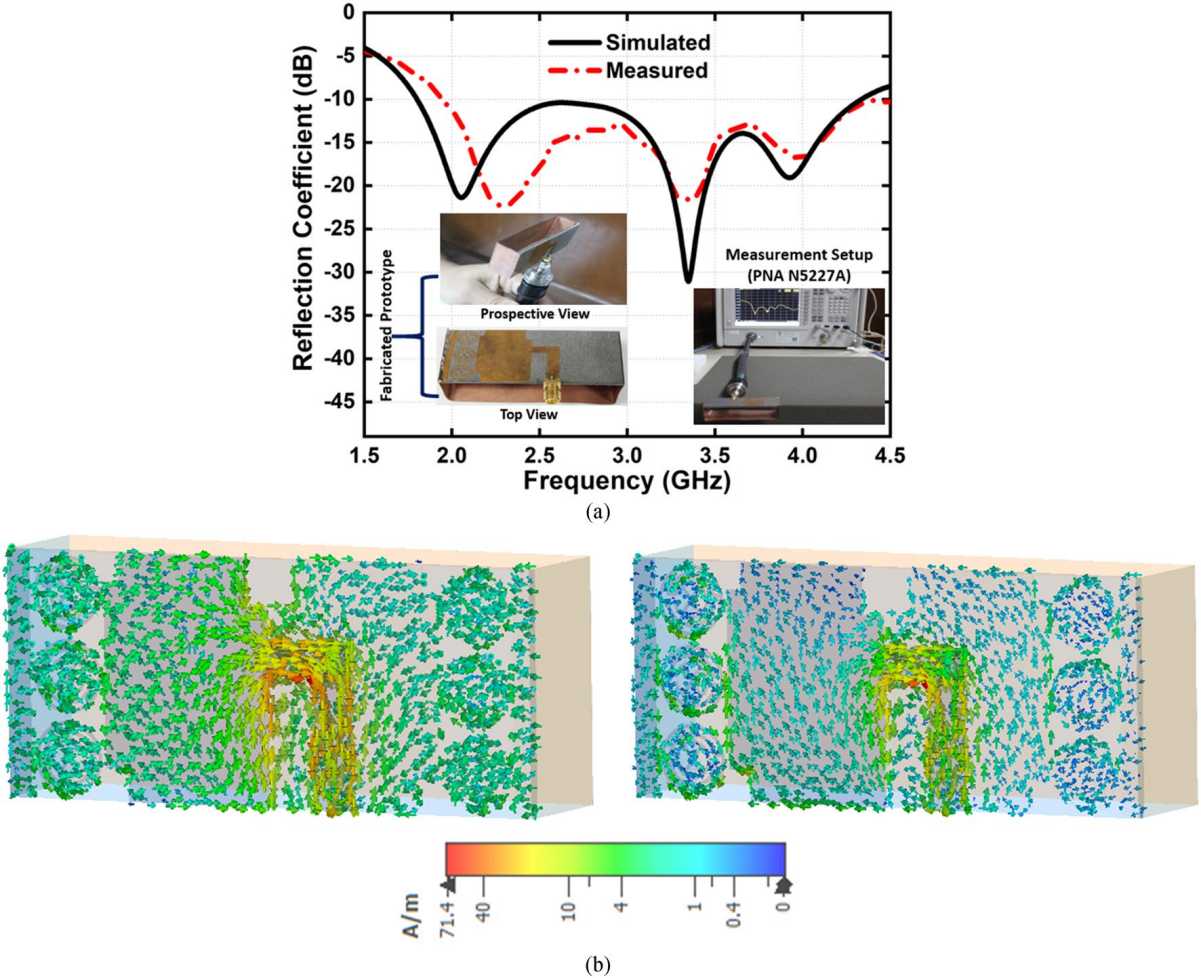
(**a**) Simulated and measured reflection coefficient and (**b**) surface current distribution at 2.1 GHz and 3.34 GHz, respectively.

Besides, the slotted dipole element has the symmetrical top and ground elements with 1 × 3 finite array MTM unit cell structure where 50Ω microstrip feeding line is used. The folded parasitic element with dipole MTM unit cell structure increases the electrical current paths, and the gaps between the patch and MTM structure are induced itself to match the input impedance. The dipole structure is responsible for the high resonant frequencies, whereas the folded structure is responsible for antenna radiation characteristics towards the unidirectionality. Also, the array of finite MTM unit cell structure has an impact of increasing the overall antenna bandwidth, efficiency and realized gain, which also ensures a significant level of compactness. An air-filled waveguide is used to characterize the individual proposed MTM unit cell structure. The incident wave propagates along Z-direction when the left and right surfaces are assigned as PEC walls, and the other two sides are assigned as PMC walls with perpendicular setup of the two waveguide ports into the Z-directions which is depicted in Fig. 2. The finite-difference electromagnetic tool CST Microwave Studio is used for the performance verification of the MTM unit cell itself and the proposed MTM-loaded 3-D antenna. Assuming, −10 dB reflection coefficient is the reference. Figure 3a represents the simulated and measured reflection coefficient of the antenna where the simulation result shows that the antenna operates over the band of 1.8 GHz to 4.34 GHz where the fractional bandwidth is approximately 83% with respect to the center frequency of 3.07 GHz.

**Figure 4:**
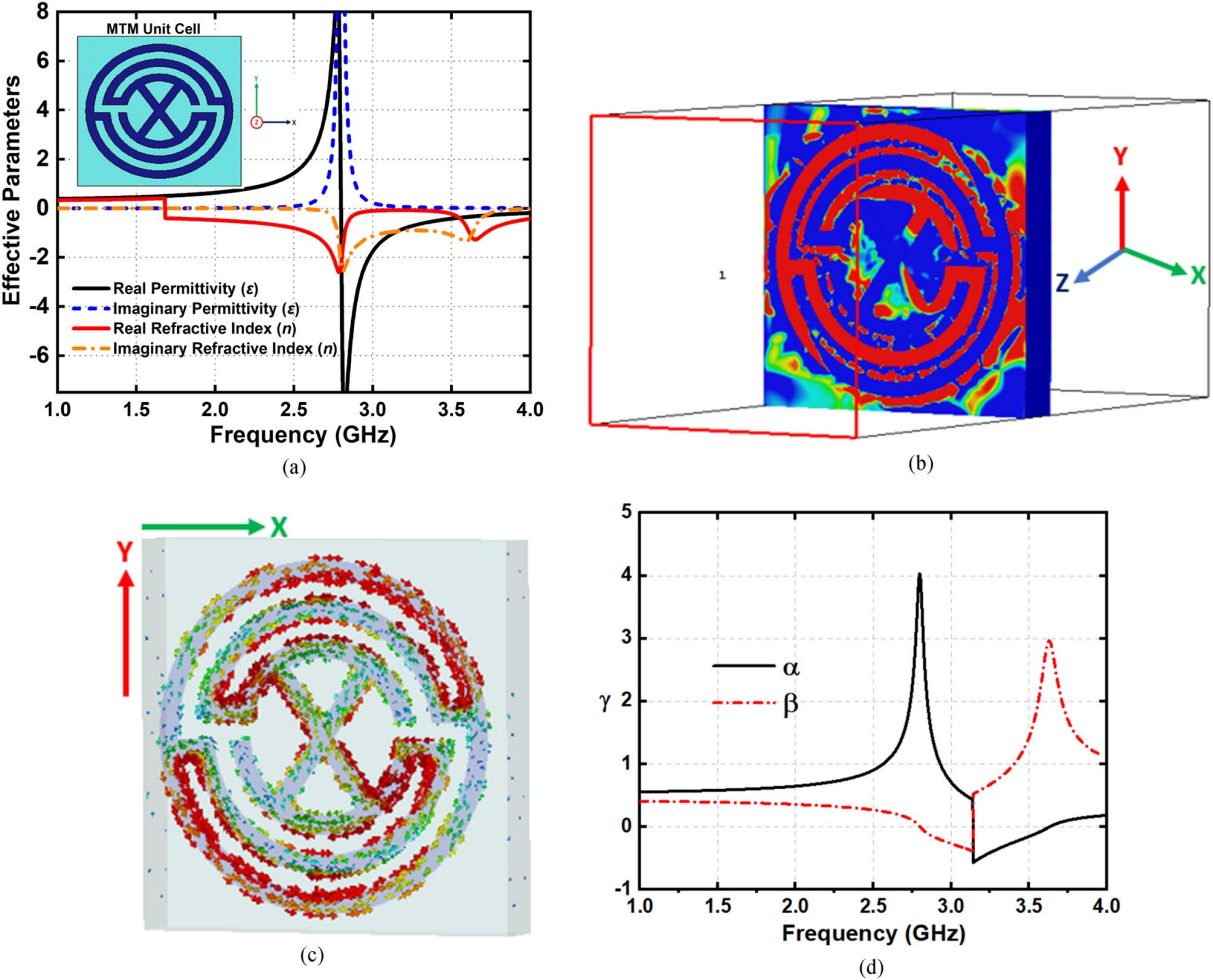
(**a**) Retrieved effective permittivity and refractive index of the MTM unit cell structure (**b**) Surface current at 2.8 GHz (**c**) E-field at 2.8 GHz, and (**d**) Propagation constant.

The measurement result indicates the approximate 80% of the fractional bandwidth with respect to the 3.22 GHz center frequency within the 1.95–4.5 GHz frequency region, which implies a good agreement between the simulated and measured results. Figure 3b represents the current distribution of the proposed antenna at 2.1 GHz and 3.34 GHz, respectively. It is observed that the fed element is considered as the main radiating element due to the maximum current concentration and circulation on the top layer. On the other hand, the ground element current distribution contributes to the radiation mechanism of the antenna. It is noteworthy that the current flows in the opposite direction to each other on the top and ground elements. Moreover, in both cases, a high front-to-back ratio can be predicted from the direction of the current flow that supports the top and ground elements and matches the impedance and directionality of the radiation characteristics. The retrieved effective permittivity and refractive index of the individual MTM unit cell structure is shown in Fig. 4, which is calculated from the scattering parameters using the Nicolson-Ross-Weir (NRW) method. The real effective permittivity and negative refractive index values are high within the frequency ranges of 1 GHz to 4 GHz, which indicates that the additional electromagnetic coupling occurs when placing the array of MTM unit cell structure in between the radiating patch and parasitic element. Besides, the array of MTM unit cell structure improves the field distribution that enlarge the antenna effective aperture.

In addition, the imaginary parts of the effective parameters approximately zero from 1 to 2.5 GHz frequency regime that indicates the loss is negligible for the MTM. Thus, the array of MTM unit cell structure within the patch and parasitic element has very strong excitement that leads to the in-band mismatch at some frequencies that creates the bandwidth extension of the antenna. Moreover, to analyze the operating mechanism of the unit cell structure, surface current and E-field distributions are presented in Fig. 4b, c. It is notable that, E-field is parallel to the unit cell surface along the X-direction, which drives the electrons to oscillate and form strong currents around the edges of the circular slots. Thus, E-field excites the proposed unit cell structure. Moreover, the currents at the edges of slots are flowing vice-versa, which form a circular current representing magnetic response. It means that the proposed unit cell structure has a strong electromagnetic (EM) coupling. In addition, it is noteworthy that the proposed unit cell structure has the ability to reflect the EM energy towards the source (Port 1). Furthermore, to analyze the wave propagation characteristics of the proposed unit cell structure, the propagation constant γ is calculated from the following Eq. (1). Attenuation constant, α and phase constant, β are obtained from the retrieved effective parameters and demonstrated as Fig. 4d. It is found that α has large values compare to β at the 1–4 GHz frequency band, which indicates the rejection of the EM wave propagation in the proposed unit cell structure^50^. This condition makes the unit cell structure suitable for applying with the antenna elements to enhance the overall performance.

1 $$\gamma = j\omega \sqrt {\mu \varepsilon } = \alpha + j\beta$$

**Figure 5:**
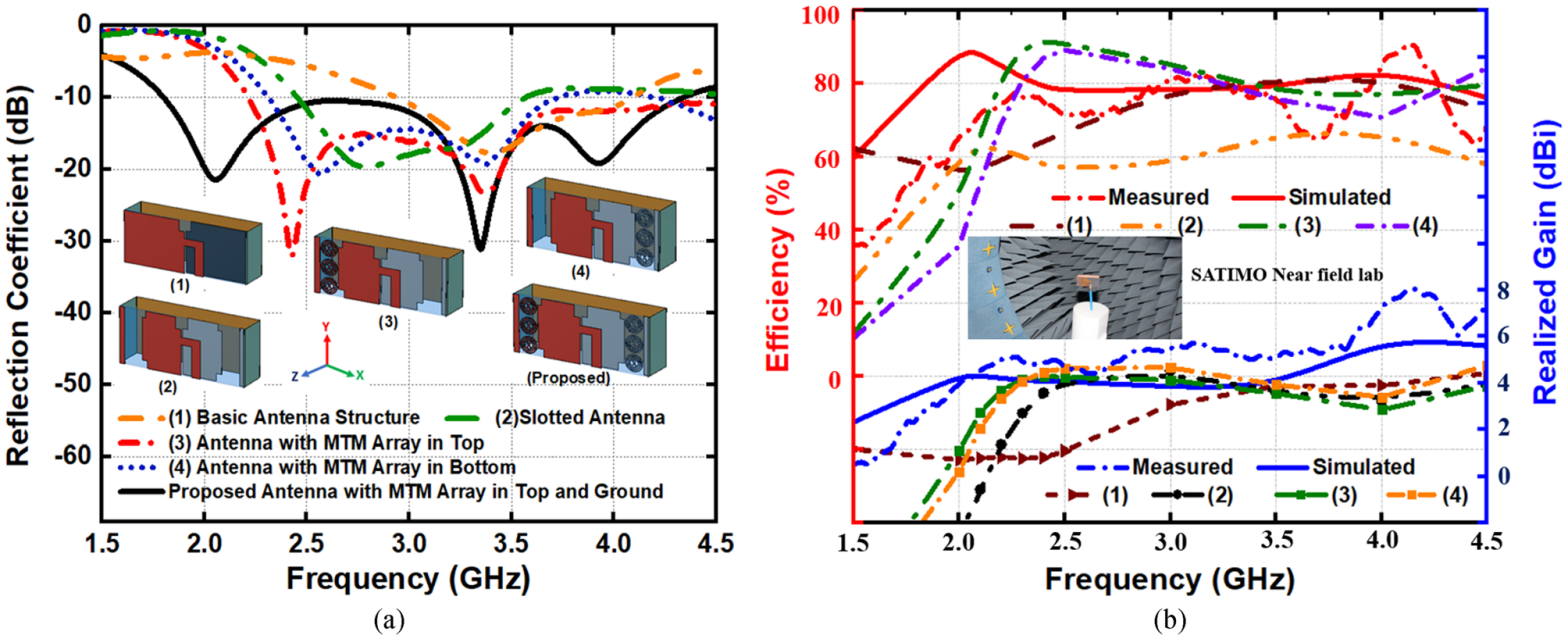
Evolution of the proposed MTM-loaded 3D-antenna (**a**) scattering parameters. (**b**) Efficiency and realized gain.

The analysis on the evolution of the proposed antenna is depicted in Fig. 5, where it represents the reflection coefficient, efficiency and realized gain of the five different structures associated with the measurement results. Figure 5a shows the analysis on changes of the antenna structures with the reflection coefficient. Antenna-1 presents the basic dipole element that generates resonance frequency at 3.5 GHz, where Antenna-2 shows the resonance at 2.7 GHz with a slotted fed and ground. The array of MTM unit cell structure is placed in between the fed and parasitic element (Antenna-3), which increases the coupling effect that lowers the resonance frequency and creates a wideband frequency range covering from 2.25 to 4.5 GHz. The Array of MTM unit cell structure is placed in between the ground and parasitic element (Antenna-4), which also shows the lowering of resonance frequency compared to Antenna-2. The proposed structure contains the array of MTM unit cell structure in both fed and ground side that shows a strong coupling effect by increasing the frequency range and lowering the resonance frequency. From Antenna-1 to the proposed antenna, all have the conducting parasitic walls connected with them. The conducting parasitic walls have a direct impact on wideband impedance matching and gain enhancement with the directional radiation pattern. Figure 5b depicts the simulated and measured efficiency and realized gain. SATIMO near field measurement lab is used to analyze the efficiency, gain, and radiation characteristics.

**Figure 6:**
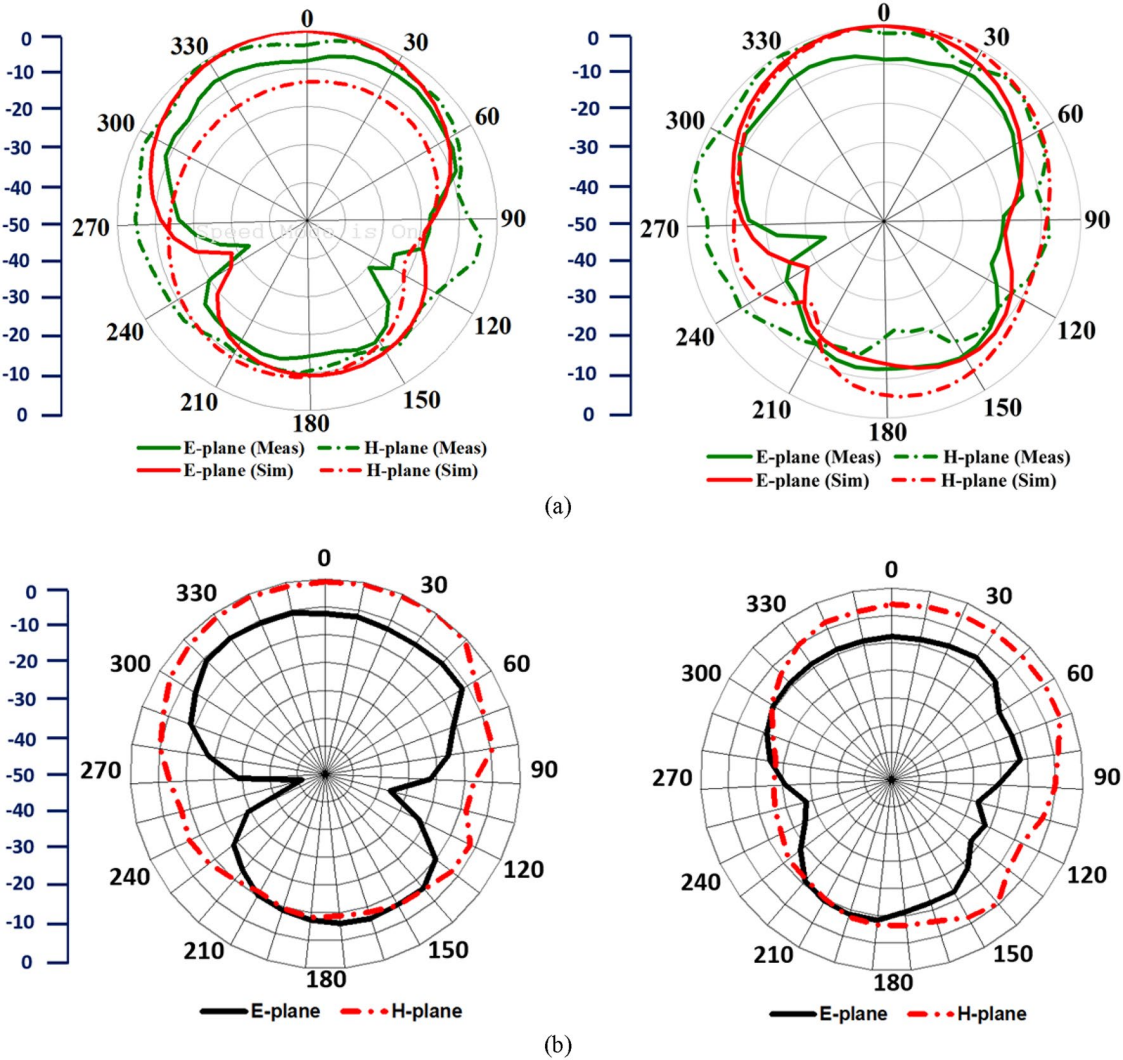
(**a**) Simulated and measured E-plane and H-plane (far-field), and (**b**) Measured E-plane and H-plane (near-field), at 2.2 GHz and 3.34 GHz, respectively.

The simulated antenna shows 89.6% and 78.5% efficiency with 4.34 dBi and 3.36 dBi realized gain at 2.1 GHz and 3.34 GHz, respectively, whereas it shows 71.7% and 77.9% efficiency with 4.52 dBi and 5.18 dBi realized gain at the same frequencies. The maximum efficiency and gain achieved from the measured results are 87.3% and 7.22 dBi, respectively, within 1 GHz to 4 GHz frequency regime. It is noticeable that the array of MTM unit cell structures increase the maximum efficiency from 66% to 89.6% and maximum realized gain from 4.55 dBi to 6.01 dBi, respectively. Figure 6a. depicts the far-field radiation characteristics of the antenna with E (XZ) and H (YZ)-plane patterns at 2.1 GHz and 3.34 GHz, respectively. The analysis shows that the simulated and measured radiation patterns have a good match as the antenna has a stable directional radiation pattern with a boresight direction with an average gain of 3 dBi along with Z-axis. It is noticeable that the H-plane has wider beamwidths compare to the E-plane radiation pattern, and the cross-polarization level is more than 10 dB in both of the planes along the Z-directions, which indicates the high polarization purity. In addition, the antenna attains an average 10.5 dB of front to back ratio along with the Z-axis. As the antenna is placed in close proximity to the head model, it is important to analyze the near-field performance. Figure 6b depicts the measured near-field radiation performance at 2.1 GHz and 3.34 GHz, respectively. Notably, a directional manner is found during the antenna transmission, which ensures the low noise from the unwanted backward direction. In addition, this directionality also enhances the range of the received scattered signal from the targeted source in the front direction that ensures less artefacts incurred by the noise.

**Figure 7:**
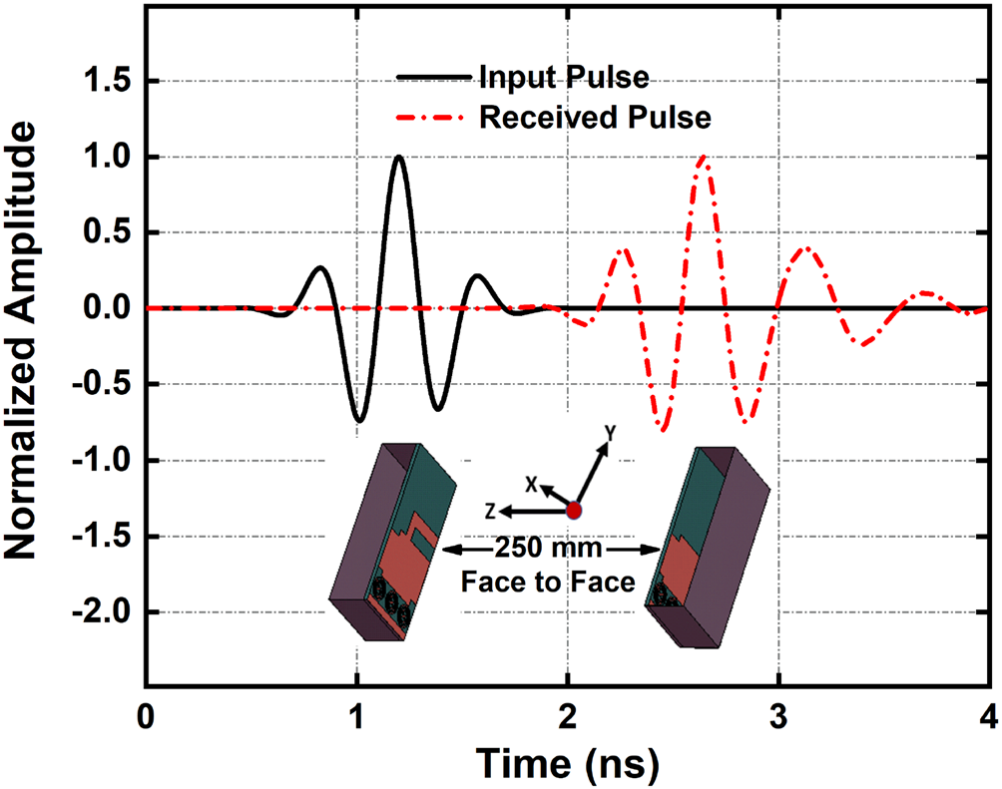
Time-domain performance: Input and received pulse.

Furthermore, the time-domain performance of the proposed antenna in terms of input and receive pulse is presented in Fig. 7 where the default CST Gaussian input signal is used to excite the antenna. Two antennas are placed at a distance of 250 mm in free space. It is noticeable that the received pulse *y(t)* is almost similar to the input pulse *x(t)* and the pulse signal shows very low distortion in minimum timing because of the high directivity of the antenna. The fidelity factor (FF) is also calculated to determine the level of signal distortion and to validate the correspondence between input and receive signals^51^. The antenna demonstrates 83% of the FF in the face-to-face direction that ensures the low distortion of the signals.

2 $$FF = \max \left( {\frac{{\int\limits_{ - \infty }^{ + \infty } {x(t)y(t - \tau )dt} }}{{\sqrt {\int\limits_{ - \infty }^{ + \infty } {|x(t)^{2} |dt\int\limits_{ - \infty }^{ + \infty } {|y(t)^{2} |dt} } } }}} \right)$$

### Mathematical modelling of the proposed antenna

The proposed MTM loaded 3D antenna mathematical modelling starts with developing the equivalent circuit by following the transmission line principle^52^. Noteworthy, all the components have been approximated by following the standard lumped element parameter equations mentioned in^53, 54^. For the main patch architecture of the proposed antenna, LC tank circuit branches are considered. Moreover, series LC networks are assumed for the horizontal and vertical edges of the PEC panels. It is noted that the basic patch structure of the proposed MTM loaded 3D antenna shows a mirror-symmetry along with Y-axis for the top and bottom edges. In that case, the input impedance (Z_0_) could be considered as Eq. (3), where it depends on the characteristics impedance Z_L_, length *l* and propagation constant γ, respectively.

3 $$Z_{in} = Z_{0} \frac{{Z_{L} + Z_{0} \gamma l}}{{Z_{0} + Z_{L} \gamma l}}$$

Furthermore, the inductance and capacitance are considered based on the following equations, where patch width (w), conducting layer thickness (t), and a correction factor (Kg) depend on the three factors such as substrate width and thickness, the dielectric constant of the dielectric film (ε_rd_), and distance between mutual overlapping strip line (d), respectively^55^.

4 $$L(nH) = 2 \times 10^{ - 4} l\left[ {\ln \left( {\frac{l}{w + t}} \right) + 1.193 + 0.2235\frac{w + t}{l}} \right]K_{g}$$

5 $$C(pF) = \frac{{10^{ - 3} \varepsilon_{rd} wl}}{36\pi d}$$

**Figure 8:**
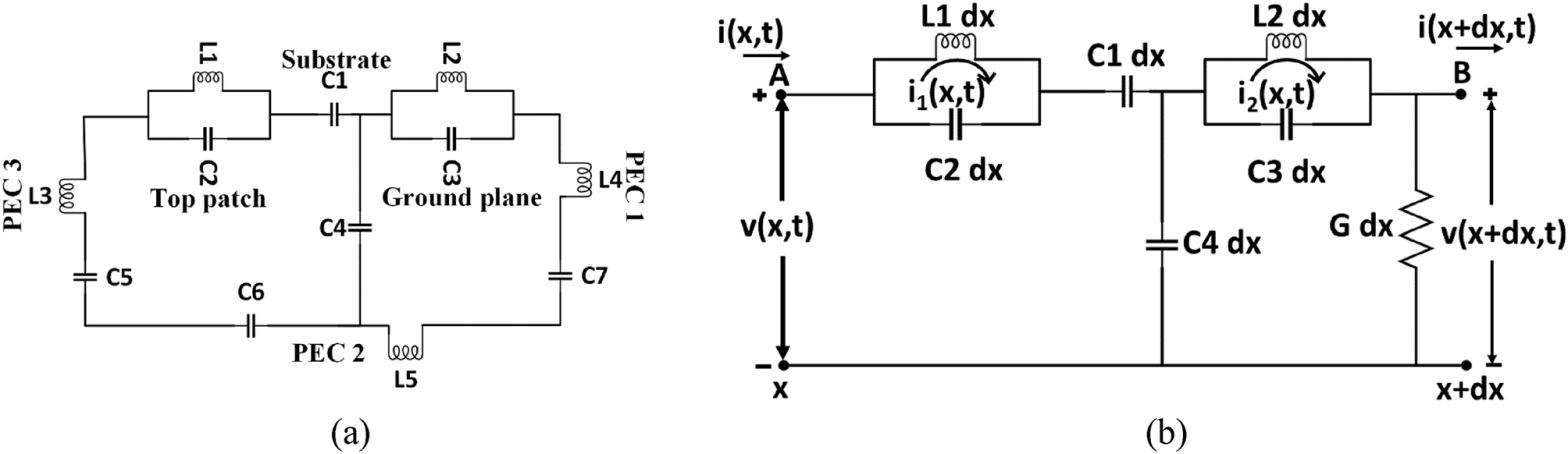
Analytical representation of 3D antenna (**a**) equivalent circuit of the 3D antenna (**b**) simplified equivalent circuit of 3D antenna for mathematical modelling.

The mathematical modelling for the MTM unit cell structures integrated with the 3D antenna is then simplified based on the transmission line principle. Figure 8a shows the equivalent circuit model where L1, C2, L2, and C3 represent the top and ground layers. Substrate and the air gap of the 3D antenna structure equivalence to C1 and C4 capacitor, respectively. The copper plate parasitic elements along with horizontal and vertical sections are characterized by three consecutive LC series circuits from L3 to L5 and C5 to C7, respectively.

For mathematical modelling, the simplified equivalent circuit has been considered by following the lumped element Transmission line principle^56–58^ as shown in Fig. 8b. Besides, the impact of EM field contribution from the parasitic element is minimal compared to the patch element, and hence, the simplified model ignores it. Besides, the voltage-drop across the inductor and the capacitor generally expressed as:

6 $$v_{L} = - L\frac{di}{{dt}}\;{\text{and}}\;v_{c} = \frac{1}{c}\int {idt}$$

Here, the considerations are: initial voltage is *V*_*i*_(x,t), initial current i(x,t), capacitance including ground plane C1 dx to C4 dx, inductance L1 dx and L2 dx, and the conductance *G* dx. Differential length (*dx*) is assumed across the x-axis.

According to feeding or excitation, voltage to the antenna *V* (x,t) at point A and consumed voltage in the entire antenna *V*_*consume*_ (x,t) would be equal to the differential voltage at point B, with representing *V* (x + dx,t). So,

7 $$V(x,t) + V_{consume} (x,t) = V(x + dx,t)$$

Elsewise,

8 $$V(x + dx,t) - V(x,t) = - \left[ {\left[ { - L_{1} dx} \right]\frac{{di_{1} }}{dt} + \frac{1}{{c_{1} }}\int {i_{1} dt + \frac{1}{{c_{4} }}} } \right.\int {i_{1} dt + } \left. {\left[ { - L_{2} dx} \right]\frac{{di_{2} }}{dt}} \right]$$

Taking the *dx → *0, from Eq. (7) and doing partial differential equation with respect to *x* we get,

9 $$\frac{\partial v}{{\partial x}} = L_{1} \frac{{\partial i_{1} }}{\partial t} - \frac{{i_{1} }}{{L_{1} C_{2} }} - \frac{i}{{C_{1} }} - \frac{i}{{C_{4} }} - L_{2} \frac{{\partial i_{2} }}{\partial t} - \frac{{i_{1} }}{{L_{2} C_{3} }}$$

For 3D antenna, terminal B current must be equal to current at A minus the current pass-through entire patch and current flows through capacitors and conductance *G.* Thus,

10 $$\frac{\partial i}{{\partial x}} = - G{}_{v} - i_{{1L_{1} }} - i_{{1C_{2} }} - i_{{2L_{2} }} - i_{{2C_{3} }}$$

As we know, the current through the lumped capacitor and the inductor is

11 $$\left. \begin{gathered} i_{{1C_{2} }} = c_{2} \frac{{\partial v_{{c_{2} }} }}{\partial t} \hfill \\ i_{{1C_{3} }} = c_{3} \frac{{\partial v_{{c_{3} }} }}{\partial t} \hfill \\ v_{{c_{2} }} = - L_{1} \frac{{\partial i_{{1L_{1} }} }}{\partial t} \hfill \\ v_{{c_{2} }} = - L_{2} \frac{{\partial i_{{2L_{2} }} }}{\partial t} \hfill \\ \end{gathered} \right\}$$

From Eq. (11),

12 $$v = - \frac{1}{G}\frac{\partial i}{{\partial x}} - \frac{1}{G}i_{{1L_{1} }} - \frac{1}{G}i_{{1c_{2} }} - \frac{1}{G}i_{{2L_{2} }} - \frac{1}{G}i_{{2C_{3} }}$$

Then again, Eq. (9) Differentiate with respect to *x*

13 $$\frac{{\partial^{2} v}}{{\partial x^{2} }} = L_{1} \frac{{\partial^{2} i_{1} }}{\partial x\partial t} - \frac{1}{{L_{1} C_{2} }}\frac{{\partial i_{1} }}{\partial x} - \frac{1}{{L_{1} C_{4} }}\frac{\partial i}{{\partial x}} - L_{2} \frac{{\partial^{2} i_{2} }}{\partial x\partial t} - \frac{1}{{L_{2} C_{3} }}\frac{{\partial i_{2} }}{\partial x}$$

and partial differential of Eq. (12), we get

14 $$\frac{\partial v}{{\partial t}} = - \frac{1}{G}\frac{{\partial^{2} i}}{\partial x\partial t} - \frac{1}{G}\frac{{\partial i_{{1L_{1} }} }}{\partial t} - \frac{1}{G}\frac{{\partial i_{{1C_{2} }} }}{\partial t} - \frac{1}{G}\frac{{\partial i_{{2L_{2} }} }}{\partial t} - \frac{1}{G}\frac{{\partial i_{{2C_{3} }} }}{\partial t}$$

Now using the relationship of Eqs. (11) and (13), we can conclude that,

15 $$\left( {\frac{1}{{GL_{1} }} + \frac{1}{{GL_{2} }}} \right)\frac{{\partial^{2} v}}{{\partial x^{2} }} = - \frac{{c_{2} }}{G}\frac{{\partial^{2} v}}{{\partial t^{2} }} - \frac{{c_{3} }}{G}\frac{{\partial^{2} v}}{{\partial t^{2} }} - \frac{\partial v}{{\partial t}} + \frac{v}{{L_{1} L_{2} G}}$$

Equation (15) is a one-dimensional partial differential equation for the proposed MTM loaded 3D antenna. It addresses the computational parameter of the 3D antenna to estimate the power or voltage requirement more precisely for sophisticated applications like EM imaging. However, the MTM unit cell incorporated in the antenna has been chosen to expedite the mutual coupling between the intra-unit cell and antenna patches at the top layer. The unit cell has a complete circle, and two mirror-reflexed gaps coupled circle shape, patch circumference, and lumped element equivalent circuit represent an LC tank circuit. Typical LC tank circuit resonance $f_{r} = \frac{1}{{2\pi \sqrt {LC} }}$, where *L* and *C* are calculated following the microstrip circuit design principle. According to the microstrip transmission line^57–59^, the simulated inductance is 2.39*nH* and capacitance is 1.385*pF.*

Corresponding transmission coefficient *f*_*r*_ evaluated as 2.78 GHz with −52 dB magnitude. So, the single unit cell MTM with such magnitude is likely to enhance the overall antenna response. Moreover, the E field response in the 3D antenna reveals that excitation through the SMA port gradually passes through the entire patch and gets significant couplings while placing between the patch and parasitic elements. Hence, identifying the three-unit cell in proximity optimizes the antenna performance and attains the maximum coupling. Moreover, to get the better approximation regarding the voltage estimation or power consumption, the following equation is considered, where Z_C_(x) and y(x) are the characteristics impedance and admittance of x in the transmission line, respectively.

16 $$\frac{dY(x)}{{dx}} + \gamma (x)\left( {\frac{1}{{Z_{c} (x)}} - Z_{c} (x)Y^{3} (x)} \right) = 0$$

## Sensitivity analysis

**Figure 9:**
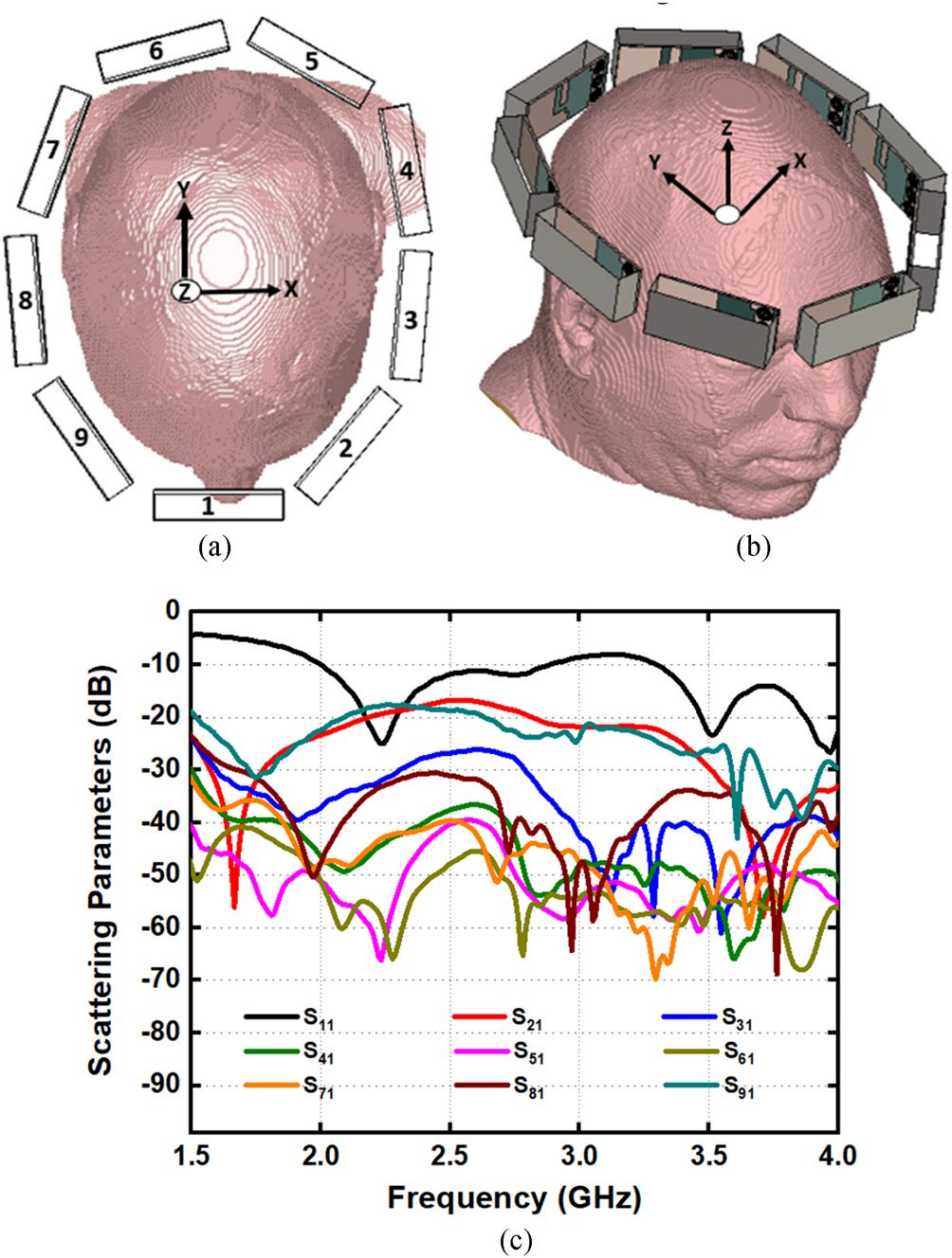
(**a**) Top view (**b**) perspective view of nine antenna setup and (**c**) antenna scattering parameters with mutual coupling effect.

**Figure 10:**
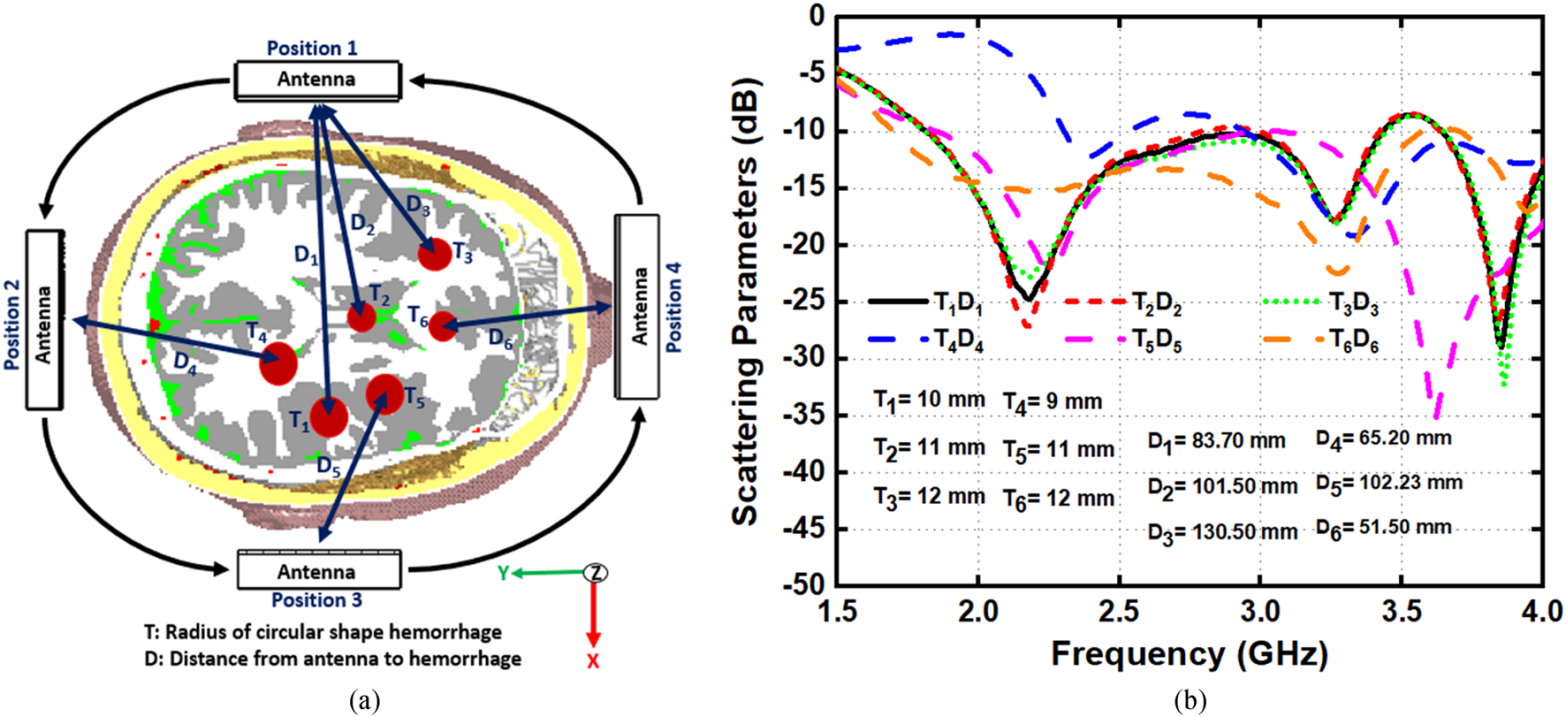
(**a**) Different antenna position with different size and location of hemorrhages and (**b**) scattering parameters.

This section analyzes the overall imaging setup in the simulation environment along with E-field, H-field, and specific absorption rate (SAR) distribution. The overall scanning setup of the imaging system with its top and perspective view are shown in Fig. 9a, b, respectively. Nine antennas are set adjacent to each other that surrounds the Hugo head model to verify the antenna performances in terms of scattering parameters and mutual coupling effects which is shown in Fig. 9c. Despite the placement with head proximity, the receiving antennas show reflection coefficient below −20 dB, which indicates the stable and comparable performance of scattering parameters and mutual coupling effects within the frequency regime. The scattering parameters in terms of different size and locations are depicted in Fig. 10a, b. Hemorrhages with six different sizes and locations have been considered analyzed with antennas that are placed in four different positions. The relative permittivity and conductivity of the region are similar to the electrical properties of blood, which are 61 and 1.79 S/m, respectively. The properties are frequency dependent, as the inserted hemorrhage is attributed to dispersive electrical properties of blood. This analysis investigates the depth sensitivity of the antenna in terms of any changes inside the brain tissue. The radius of the circular shaped hemorrhages ranges from 9 to 12 mm. The analysis shows that the responses of the scattering parameters are different compared to the free space analysis. The significant changes on scattering parameters indicate the useful possible identification of the hemorrhages with different size and locations from the scattered signals.

**Figure 11:**
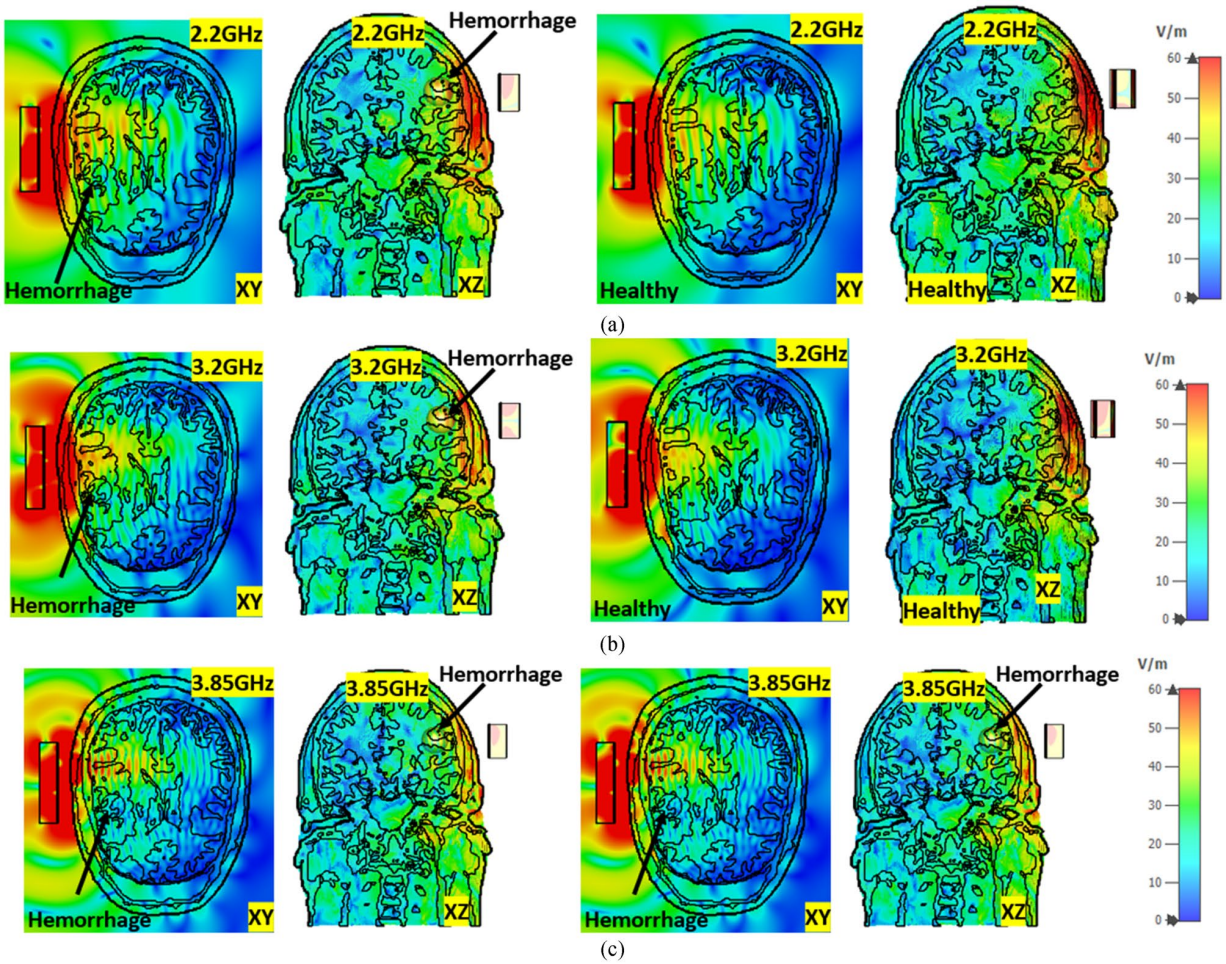
E-field distribution of healthy and unhealthy head at (**a**) 2.2 GHz, (**b**) 3.2 GHz, and (**c**) 3.85 GHz, respectively.

**Figure 12:**
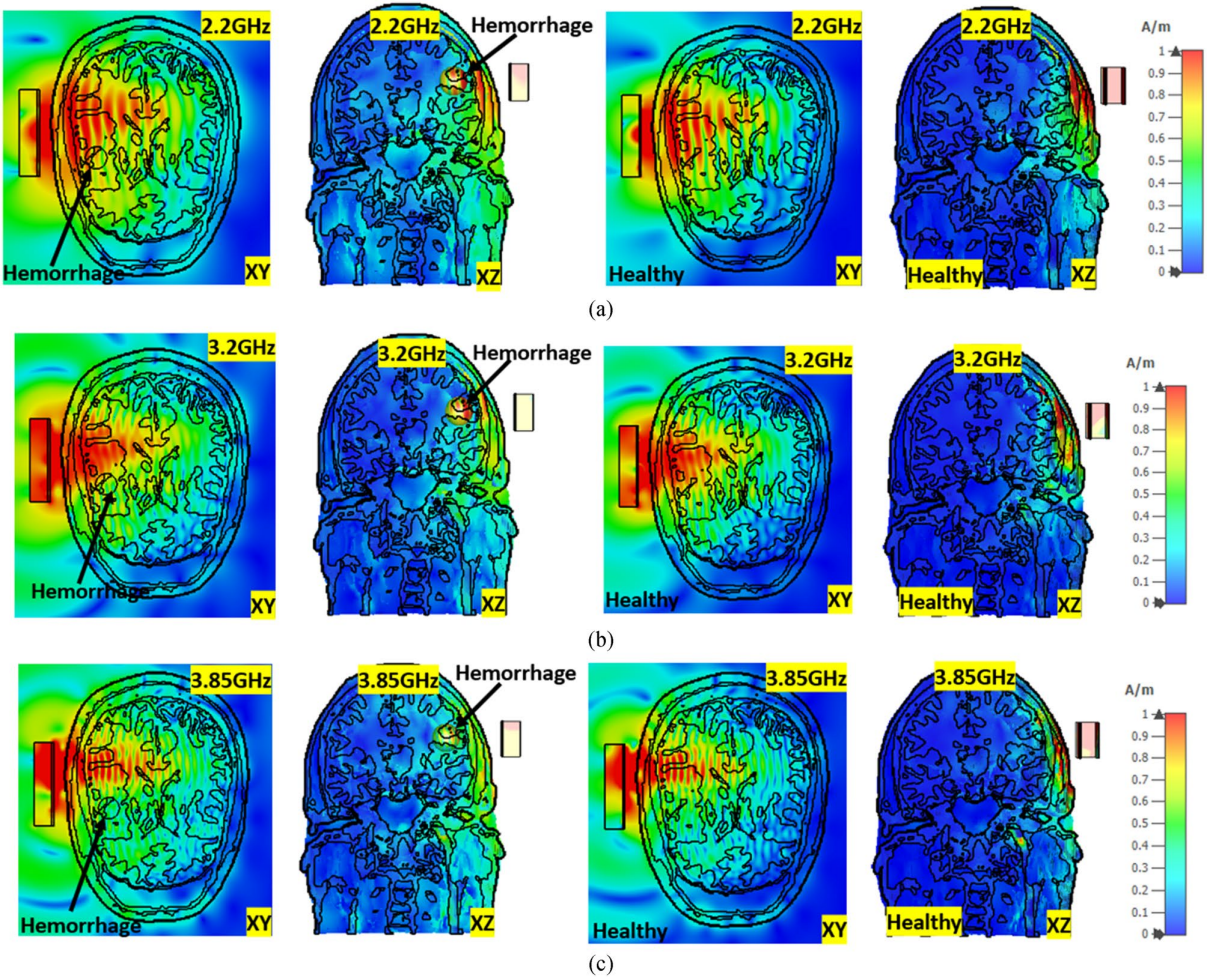
H-field distribution of healthy and unhealthy head at (**a**) 2.2 GHz, (**b**) 3.2 GHz, and (**c**) 3.85 GHz, respectively.

Figures 11a–c and 12a–c represent the E-field and H-field distributions inside the healthy and unhealthy realistic head model with and without hemorrhage at 2.20 GHz, 3.20 GHz, and 3.85 GHz, respectively. The proposed antenna shows unidirectional wave propagation towards the realistic head model, which shows the EM wave penetration inside the lossy head tissue. It is noticeable that there is a propagation difference of the E-field and H-field with and without placement of hemorrhage due to the changes in the dielectric properties and tissue surroundings. The observing fact is that E-field and H-field decrease inside the lossy head tissue. As the proposed system consists of nine antenna array elements, which cover the whole head, the wave propagation depth is sufficient for the successful image reconstruction with all conveying tissue information.

Furthermore, effective medium ration (EMR) is another important consideration in the EM imaging system that explains the antenna and its field interaction more precisely. To be more specific, EMR is defined as the ratio between operating wavelength (λ) and the dimension of the respective element. As the MTM unit cell and antenna combinedly work as a single element, the EMR becomes incomprehensible and less than one for the entire operating spectrum. The proposed antenna is intended for EM head imaging system, Maxwell–Garnett's^60, 61^ theory would be more suitable to explain the impact of the antenna.

For a human brain, majority biological tissues are anisotropic medium. Thus, the + *Z* axis propagation and a two-phase multisystem are considered. So, dielectric characteristics of brain tissues in the human body are similar to the suspension model described in Maxwell–Garnett equation^60^.

17 $$\varepsilon_{eff} = \varepsilon_{air} - 3f\varepsilon_{air} \frac{{\varepsilon_{air} - \varepsilon_{s} }}{{\varepsilon_{s} + 2\varepsilon_{air} }}$$

Here, $\varepsilon_{eff}$ is the effective permittivity of the composite structure, $\varepsilon_{air}$ and $\varepsilon_{s}$ are the permittivity of air and substrate, respectively.

18 $$\nabla \times \vec{H} = (\sigma + \omega \varepsilon^{\prime \prime } )\vec{E} + j\omega \varepsilon^{\prime } \vec{E}$$

**Figure 13:**
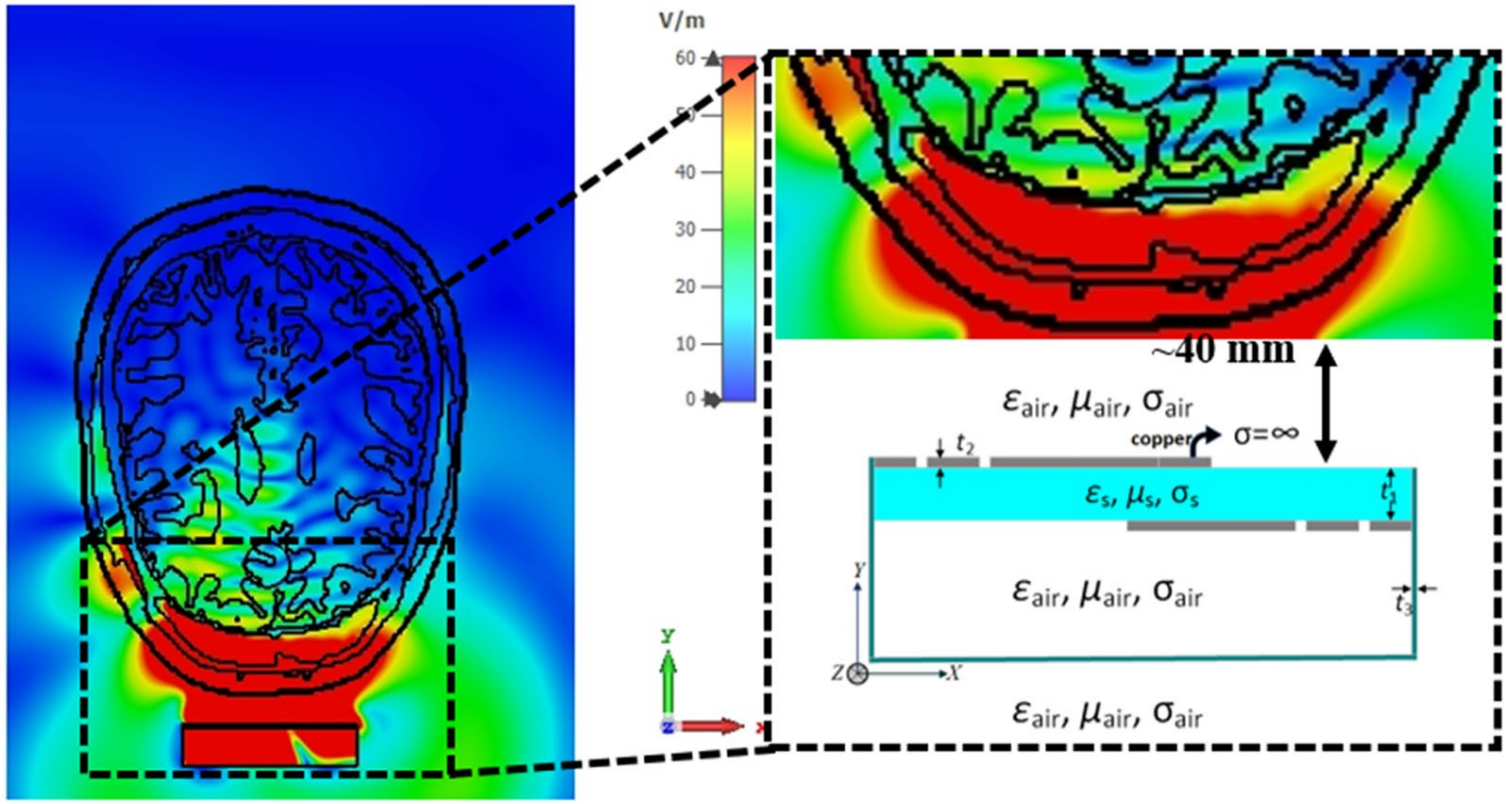
EMT perspective view for a phantom cross-section and the proposed antenna with relevant dielectric parameters.

Apart from that, conductivity ($\sigma$) and loss tangent permittivity ($\varepsilon$) depend on the travelling wave current position in any medium, as shown in Fig. 13. It represents the absorbed EM power in the structure that reflects the Ampere’s law as in Eq. (17), where the curl relation represents the magnetic field intensity $\vec{H}$(A/m) and electric field intensity $\vec{E}$ (V/m). An approximate 40 mm distance is considered between the measured phantom and the entirely arbitrary antenna. The distance is kept as close to the phantom as possible to reduce losses in air and other materials. Besides, the anisotropic medium and MTM loaded 3D antenna structure interacts with each other based on instantaneous changes between the σ and ε. So, to understand the insights of this phenomenon, the effective media acting during the imaging data extraction are analyzed. Dispersion relations must be evaluated before analyzing the medium through Effective Medium Theory (EMT). A single 3D antenna with and without MTM integration is placed for the phantom using different ‘Eigen Mode Solver’ to find the ‘first Brillouin Zone’. The amount of EM field interaction with set of points that crossing the effective medium of any ‘Bragg plane’ can be defined by this zone. Hence, the presence of MTM with the proposed antenna for imaging purpose will find whether the medium between excitation terminal to targeted area having effective interaction or not. According to average field and Effective Medium theory^61^ with the vicinity of zone dispersion mode, the wave periodic spatial characteristics are theoretically expressed as^62^ ω≈ω0 + (k2/2 m*). Here, m* is effective mass of photon that interacts with the periodic structure,  is Planck’s constant and k is wave vector. Therefore, Eq. (18) clearly represents the effective medium field nature.

**Figure 14:**
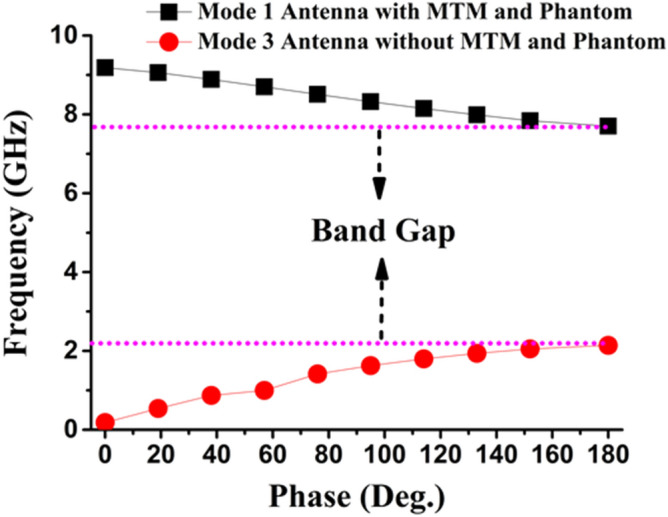
Dispersion diagram of the proposed antenna.

Moreover, the dispersion analysis performed in TE and TM mode choses the Hexahedral mesh and Jacody Davidson Method (JDM) for Mode 1 and Mode 3. These modes (1 and 3) with a certain bandgap will predict whether the excitation have a specific number of structures in lowest resonance. The simulation shows only fundamental mode (which is expected) with the lowest mode for faster calculation in JDM. Figure 14 shows the dispersion diagram where Mode 3 found the fundamental consideration of antenna without MTM and phantom. Mode 1 represents dispersive behavior with MTM and phantom. The horizontal bandgap along 0 to 140 degree is relatively high compared to 140 to 180 degree. So, introducing the MTM with the proposed 3D antenna helps to achieve the Brillouin Zone boundaries within half a path of the travelling wave. Though the bandgap is around 5.56 GHz, which is relatively high to achieve the fundamental frequency ranges, but further accurate approximation of effective medium may reduce the bandgap with different MTM design.

However, the uneven distribution of blood cells, water molecule in tissue, and vicinity of the cell membranes affect the field distribution's conductivity and permittivity. The polarization of water molecules generally expedites by tissues and other biomolecules within the phantom. Besides, blood volume fraction at instantaneous is unpredictable as the frequency changes. According to the EMT Eq. (13), the distribution shown in Figs. 11 and 12 has the characteristics of dielectric dispersion. On the other hand, the blood cell has interface polarization where field propagation changes the orientation of the external electric field from the antenna.

**Figure 15:**
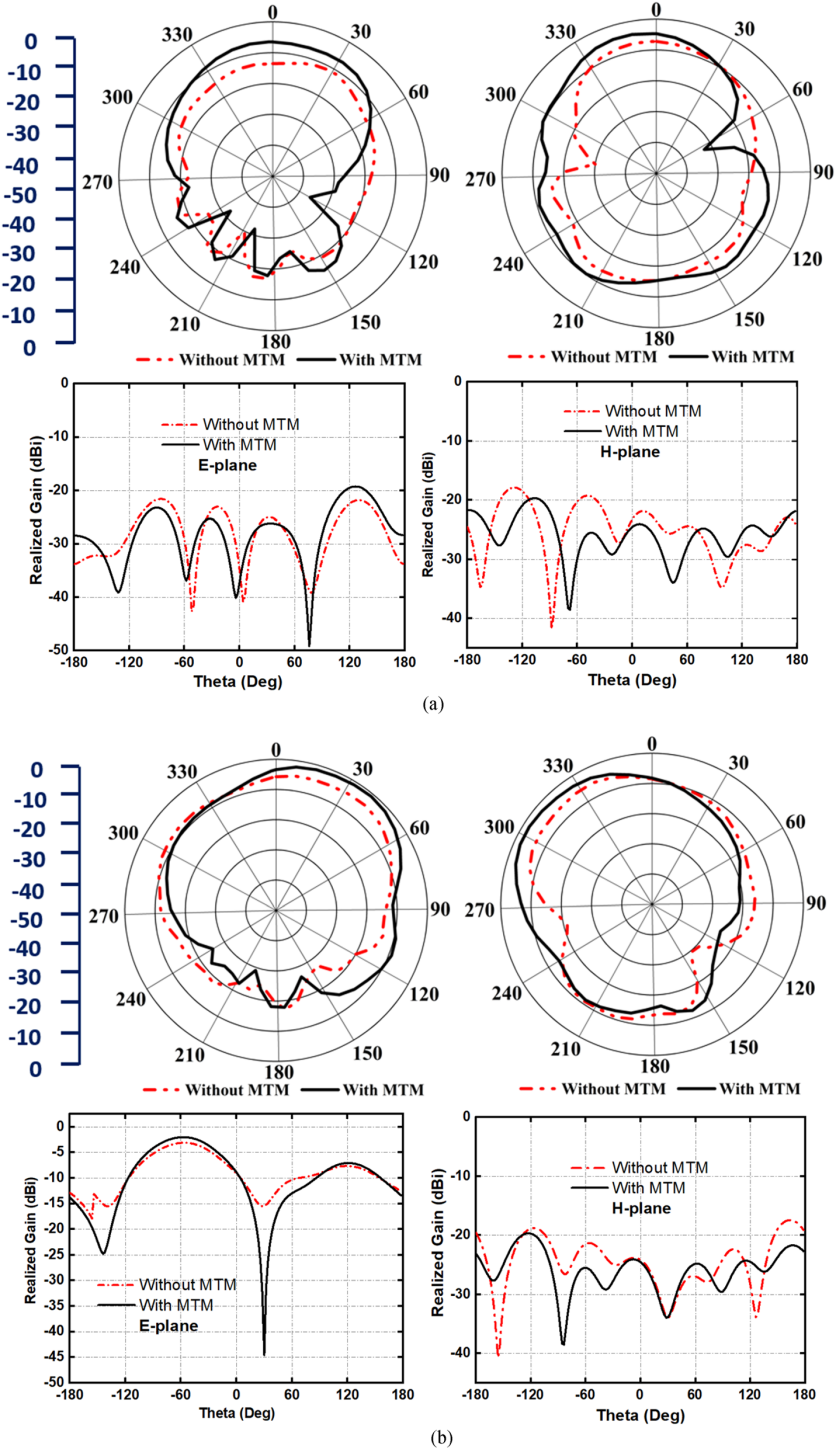

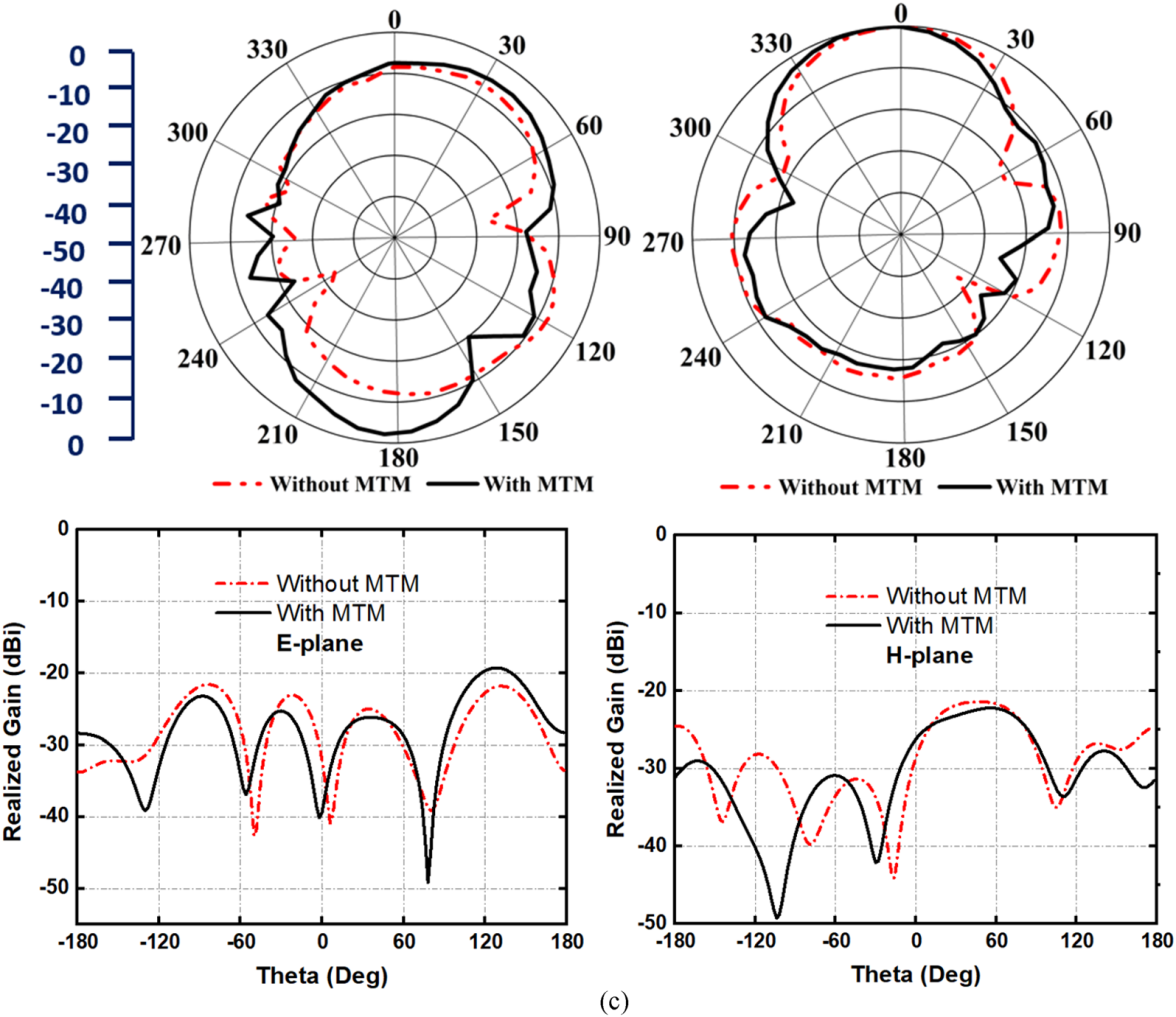
Antenna radiation and cross-polarization behavior with and without metamaterial structure in close proximity to the head phantom. E-plane and H-place at (**a**) 2.2 GHz, (**b**) 3.2 GHz, and (**c**) 3.85 GHz, respectively.

Figure 15 represents the simulated antenna radiation patterns with their cross-polarization behavior in E-field and H-field at 2.2 GHz, 3.2 GHz, and 3.85 GHz, respectively, when the antenna is placed near the head model. The analysis has been performed by applying a single antenna at a distance of 15 mm from the head model. Besides, the inclusion of MTM structure and its effect on on-body radiation characteristics are presented in this study. It is noteworthy that the MTM loaded antenna shows the maximum directionality with a reduction of back lobe radiation by filtering the MTM electromagnetic radiation compared to the antenna without MTM. To support the analysis, the cross-polarization behaviors of the antenna with and without MTM are also analyzed. Notably, the MTM improves the cross-polarization of the antenna. Although some point shows high cross-pol value without the MTM structure, it is not in the direction of high co-pol. Therefore, there will be a minor impact on the results of the imaging system due to the high cross-polarization values.

**Figure 16:**
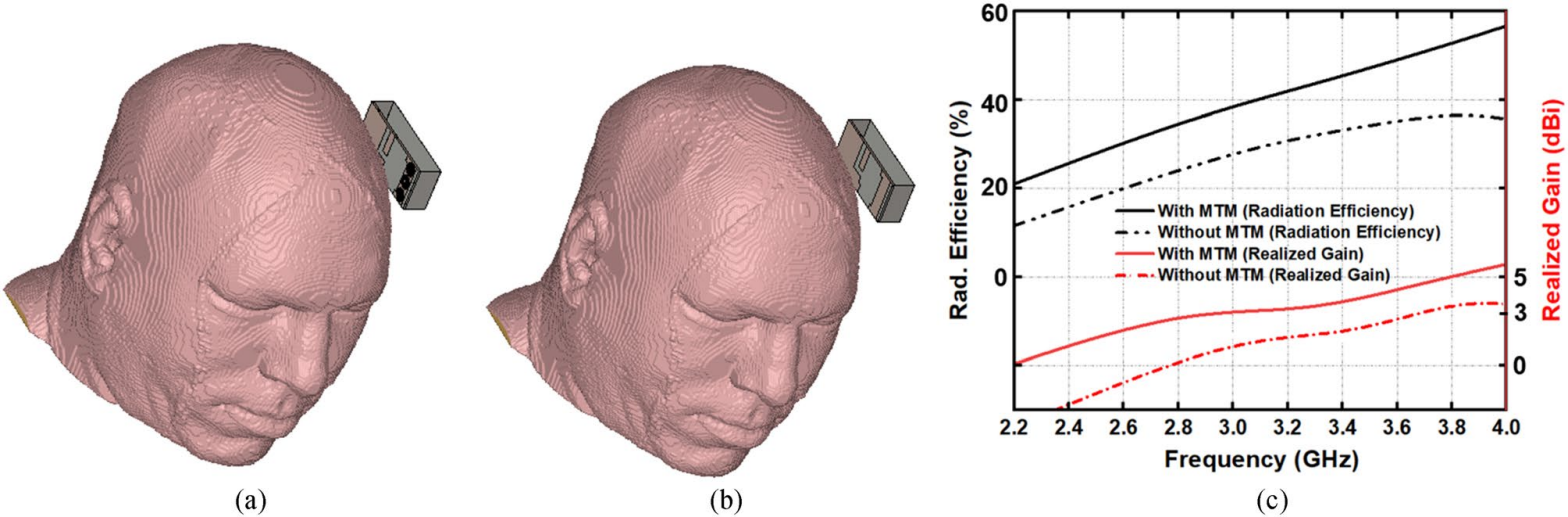
Near-field head model analysis (**a**) with MTM (**b**) without MTM and (**c**) radiation efficiency and realized gain.

Furthermore, the effect of MTM is analyzed with the head model in the near-field region. Figure 16a, b depicts the head model with and without MTM structure. The antenna is placed to the close proximity of head model and the simulation is performed by utilizing CST microwave studio. Figure 16c represents the radiation efficiency and realized gain of the antenna with and without the inclusion of the MTM structure. The MTM structures within the antenna increase the radiation efficiency and gain when placed to the head proximity. In addition, the performances in terms of radiation efficiency and gain with similar placement are presented in Table 1. It is noticeable that the MTM structures within the antenna increases the radiation efficiency and gain when placed near the head model.

**Table 1:** Realized gain and radiation efficiency of the antenna with and without metamaterial structure in proximity to the head phantom.

Frequency (GHz)	Radiation efficiency (%)		Realized gain (dBi)	
	Without MTM	With MTM	Without MTM	With MTM
2.20	11.5	18	−5.03	−2.16
3.20	30	42.8	0.42	2.40
3.85	36.7	53.8	2.50	4.97

**Figure 17:**
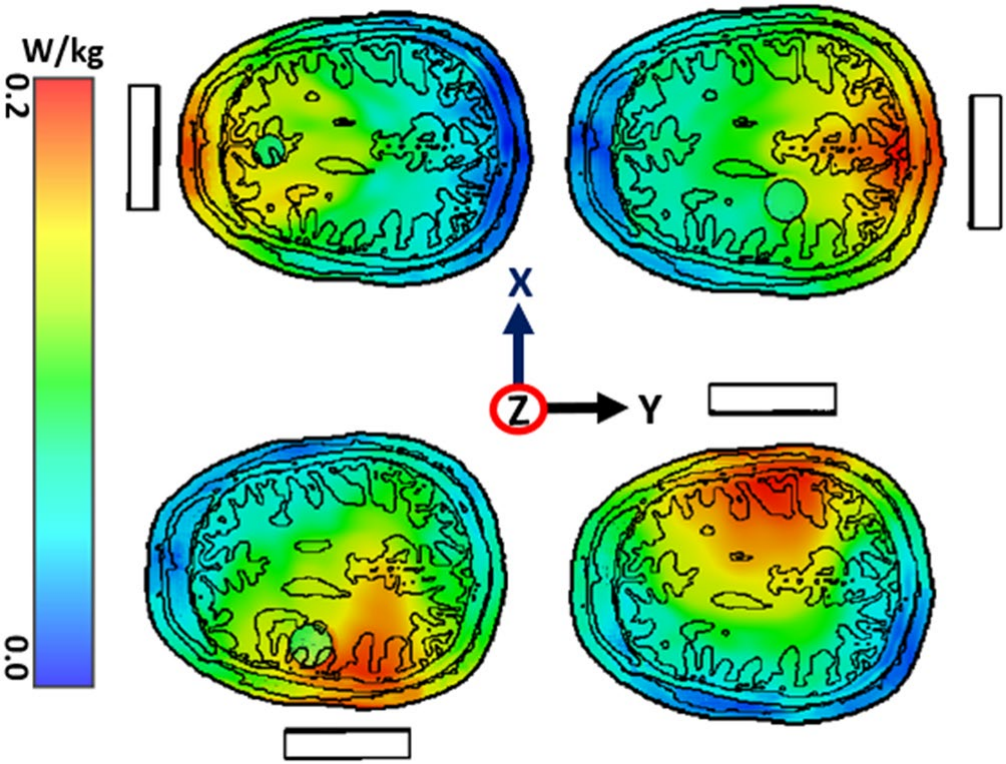
The specific absorption rate (SAR) inside the head phantom with antenna operation in different positions within XY-plane with 1mW input power at 2.20 GHz.

The SAR distribution is an important consideration to protect the human body from harmful radiation effects. As the proposed antenna is placed near to the head model, the SAR must be calculated and analyzed. The input power for the antenna is 1mW, and only one antenna acts as a transmitter at a particular time. Figure 17 represents the SAR distribution for four different positions of the antenna for 1 g of tissue at 2.2 GHz. The analysis further continues to calculate the SAR distribution for 1 g and 10 g of tissue in four different positions with different resonance frequencies as per the scattering parameters, which is shown in Table 2. Notably, 10 g tissue absorbs less electromagnetic energy compared to the 1 g of tissue. The maximum SAR positions are identified at the exterior head tissue layer. The maximum SAR positions are identified at the exterior head tissue layer. The maximum values are 0.117, 0.149, 0.161, and 0.155 of 1 g tissue, and 0.050, 0.076, 0.071, and 0.067 of 10 g tissue, respectively, for the four different antenna positions which satisfy the IEEE public radiation exposure limit of 1.6 W/kg^63^.

**Table 2:** Sar values distribution when mtm antenna is placed on different positions in xy-plane near to the head model.

Antenna position	Frequency	1 g	10 g
	2.2	0.085	0.045
	3.2	0.117	0.050
	3.85	0.070	0.030
	2	0.135	0.073
	2.2	0.118	0.062
	3.85	0.149	0.076
	2.2	0.134	0.063
	3.2	0.154	0.071
	3.85	0.161	0.065
	2.2	0.120	0.046
	3.2	0.143	0.054
	3.85	0.155	0.067

## Phantom fabrication and measurement

**Figure 18:**
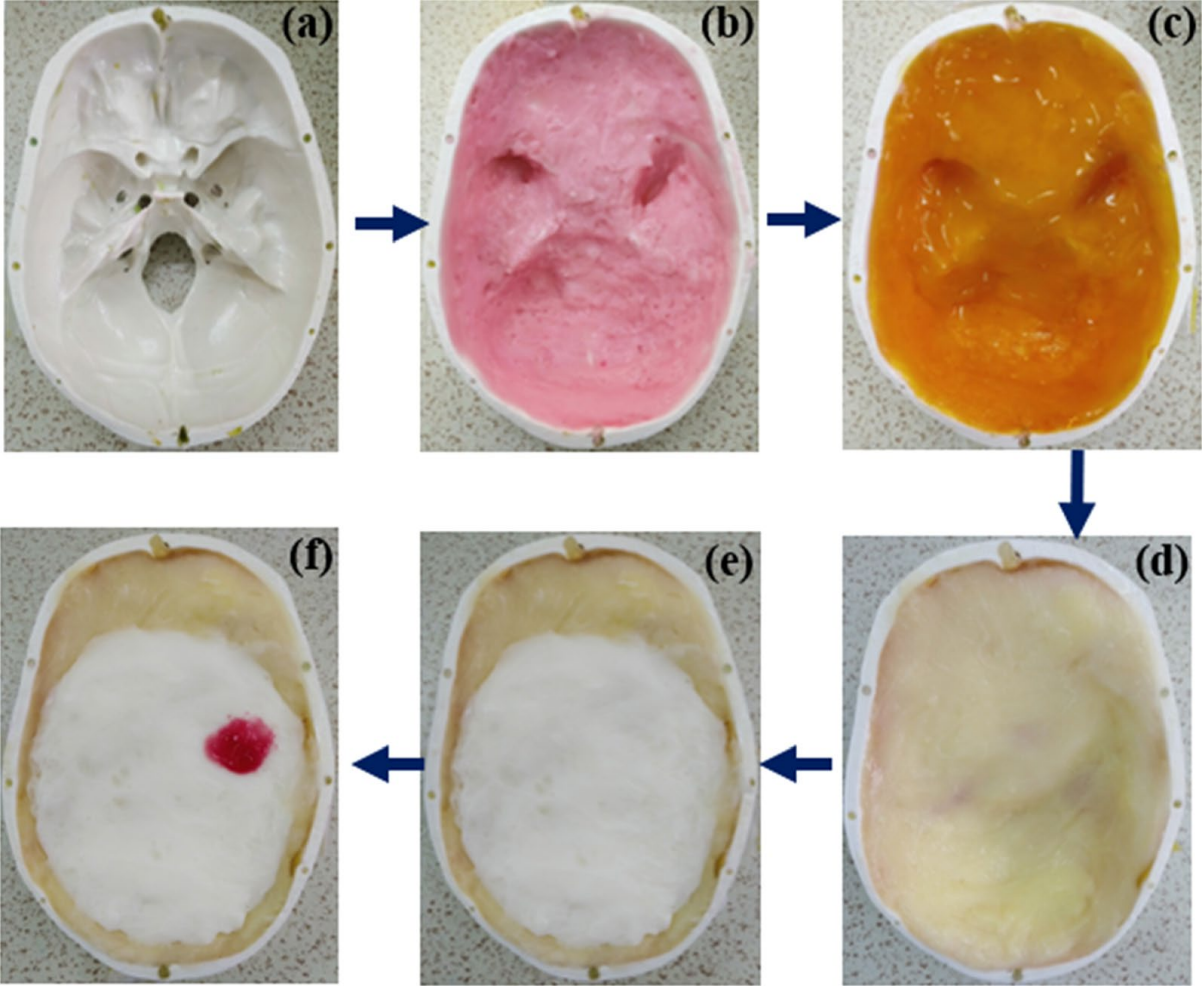
Steps of 3D head phantom fabrication. (**a**) Blank (**b**) Dura (**c**) CSF (**d**) gray matter (**e**) white matter (**f**) phantom with hemorrhage.

**Figure 19:**
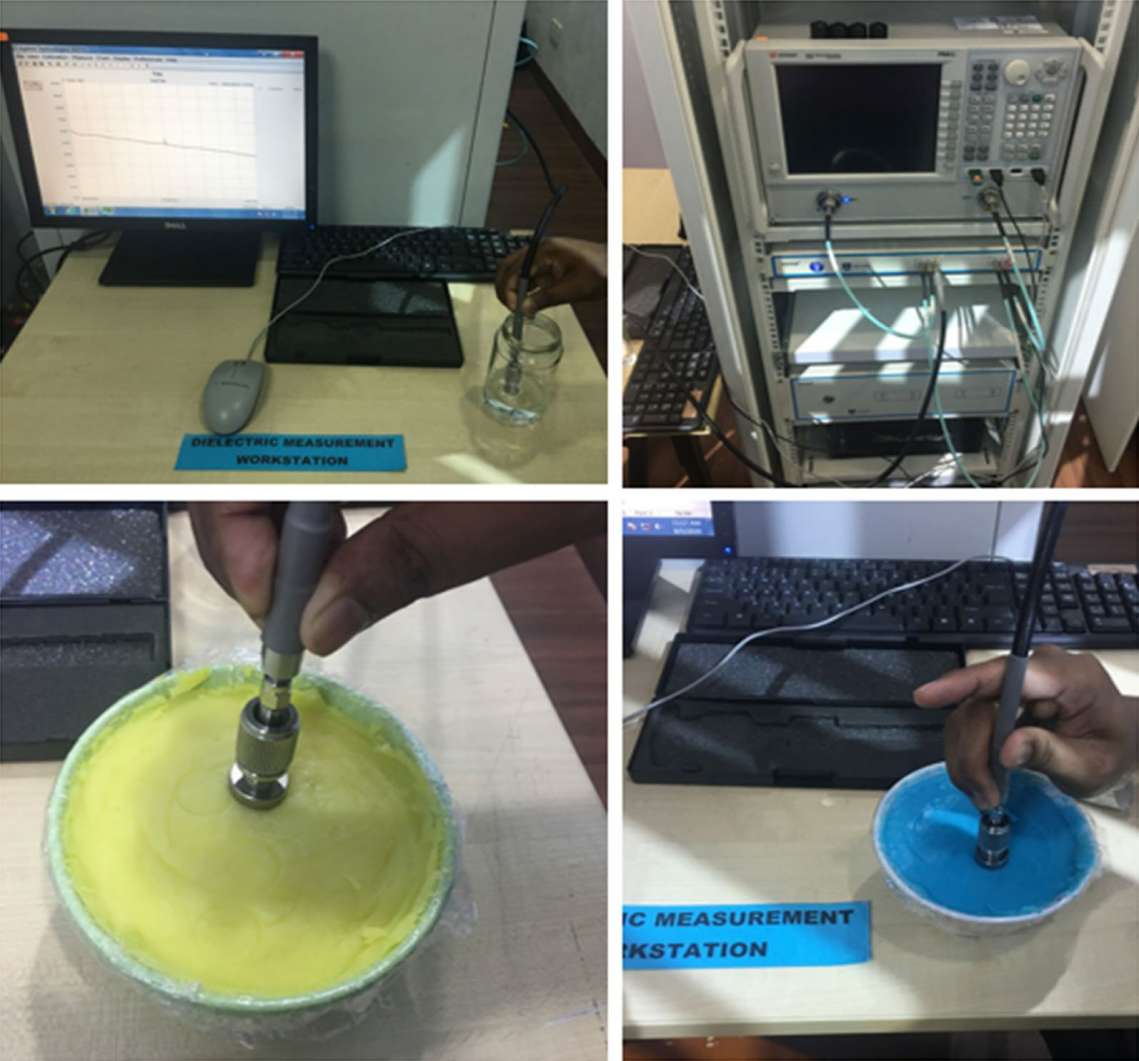
Measurement procedures of the fabricated head phantom.

**Figure 20:**
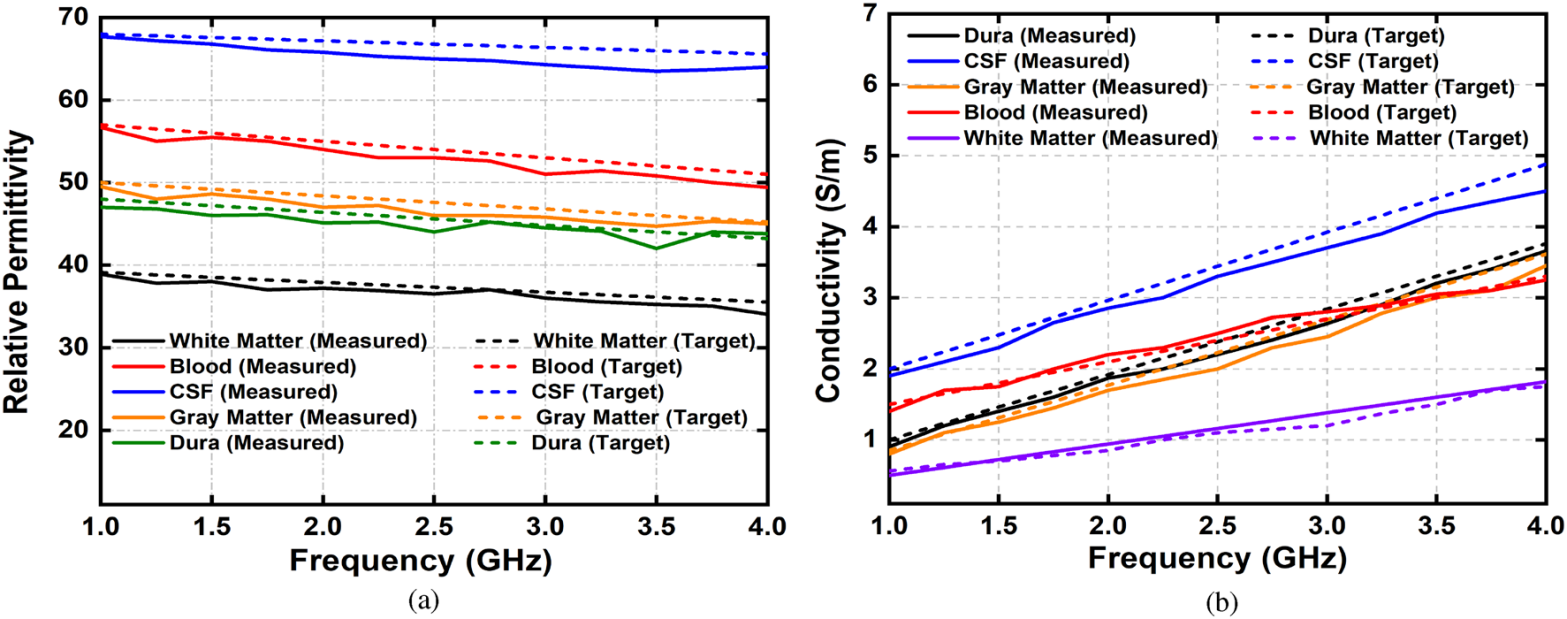
Actual and measured values of human head phantom (**a**) relative permittivity and (**b**) conductivity.

The fabrication and measurement of the heterogeneous human head phantom with five subdivided brain parts of Dura, CSF, gray matter, white matter, and blood (hemorrhage) are presented in this section. The fabrication is done according to the guidelines given in^64^. Agar, gelatin, corn flour, sodium azide, sodium chloride, distilled water, and propylene glycol are used in different compositions to fabricate the tissue-mimicking materials of the head, which is also depicted in Fig. 18 by showing the step by step adding procedure of dura, CSF, gray matter, white matter and blood (hemorrhage), consecutively. A dielectric probe kit from KEYSIGHT (85070E) which is operated by the 85,070-software installed with a network analyzer (PNA-L N5232A; 300 kHz to 20 GHz) is used to measure the electrical properties of the fabricated materials. The calibration of the probe is performed with the sterile water, and later all the material samples are measured from 300 MHz to 5 GHz frequency range. The measurement setup and material sample are shown in Fig. 19. The actual^65^ and measured relative permittivity and conductivity of the developed tissue-mimicking phantom materials are presented in Fig. 20, which demonstrate a reasonable similarity between them.

## Simulation and experimental validation with image reconstruction

**Figure 21:**
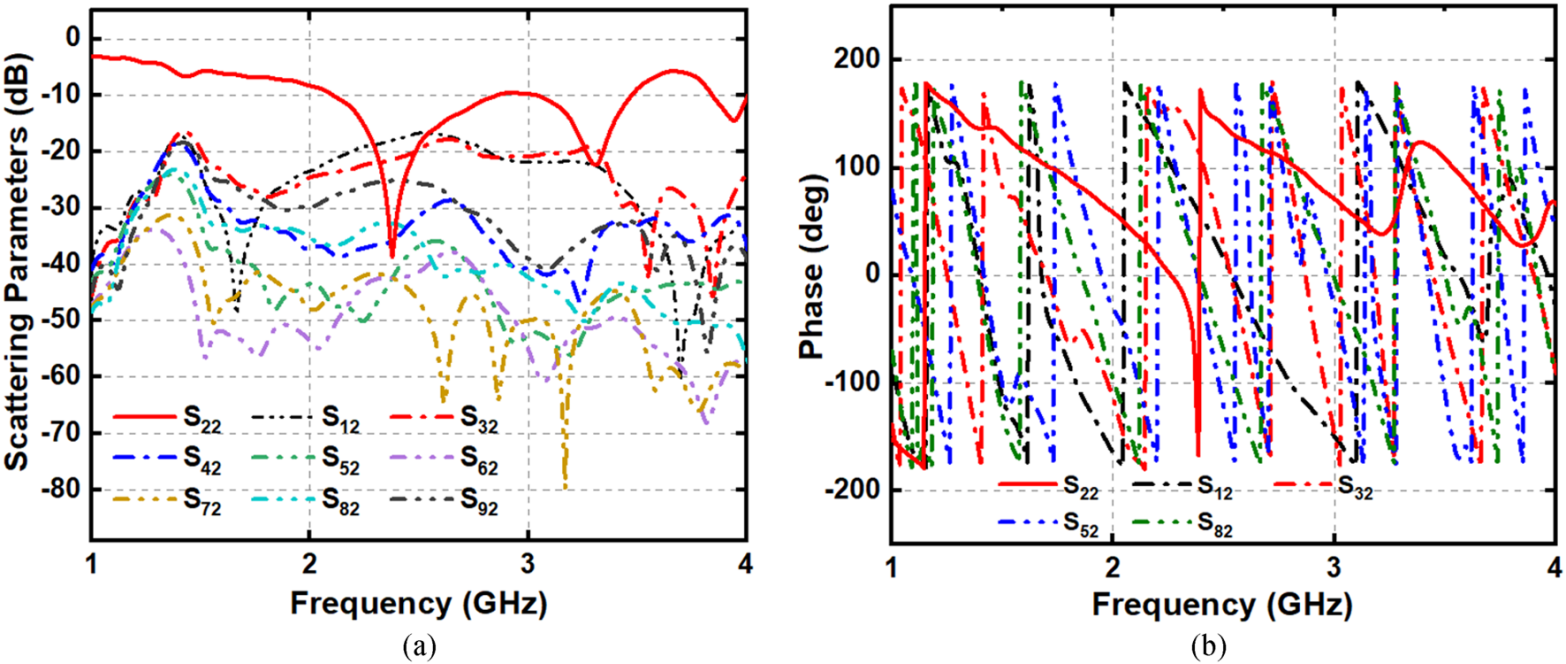
Sample (**a**) scattering parameters and (**b**) phase (antenna 2: transmitter).

**Figure 22:**
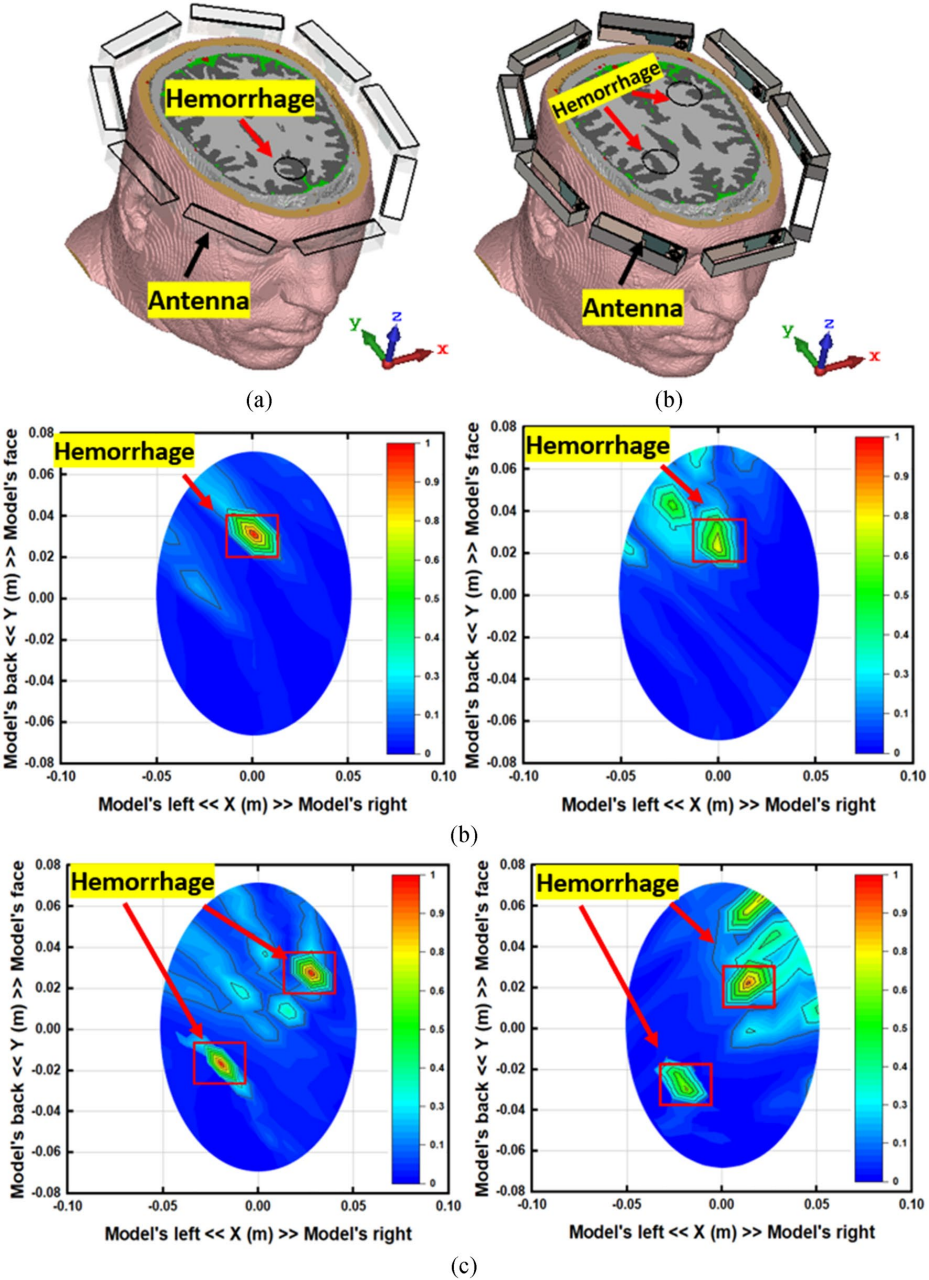
Simulated near-field image reconstruction (**a**) Perspective head model with single and double hemorrhage (**b**) single hemorrhage detection with and without MTM loaded antenna (**c**) double hemorrhage detection with and without MTM loaded antenna.

The image reconstruction analysis for the simulated head model in the near-field region is presented in Figs. 21 and 22. The Hugo head model is utilized in CST microwave studio to analyze the detection performance. The analysis is performed in two steps; first, a single hemorrhage is inserted into the head model, and images are reconstructed by applying nine antenna array with and without MTM unit cell structures. Secondly, two hemorrhage targets are inserted into two locations of the head model and the image reconstruction follows a similar manner as single one. Blood, skin, fat, CSF, grey and white matter are used in the simulated head model. The hemorrhage mimics the electrical properties of the head. In both cases, the operating bandwidth is considered 1 to 4 GHz. The sample scattering parameters and phase are depicted in Fig. 21, when antenna 2 acts as a transmitter. The image reconstruction procedure starts with collecting the scattering parameters and phase of the antenna. In the simulation, one antenna acts as a transmitter and the other eight antennas act as the receiver. The data are collected from each antenna simultaneously and continued for the image processing. The IC-CF-DMAS algorithm is also analyzed and applied based on the signal contrast to reconstruct the images. It is noteworthy to mention from Fig. 22 that the antenna can detect the single and double hemorrhage by utilizing both with and without MTM scenario. Moreover, it is important to consider that a better localization of the targets and a reduction of the artefacts are observed from the analysis when the MTM structure is added to the antenna prototype.

**Figure 23:**
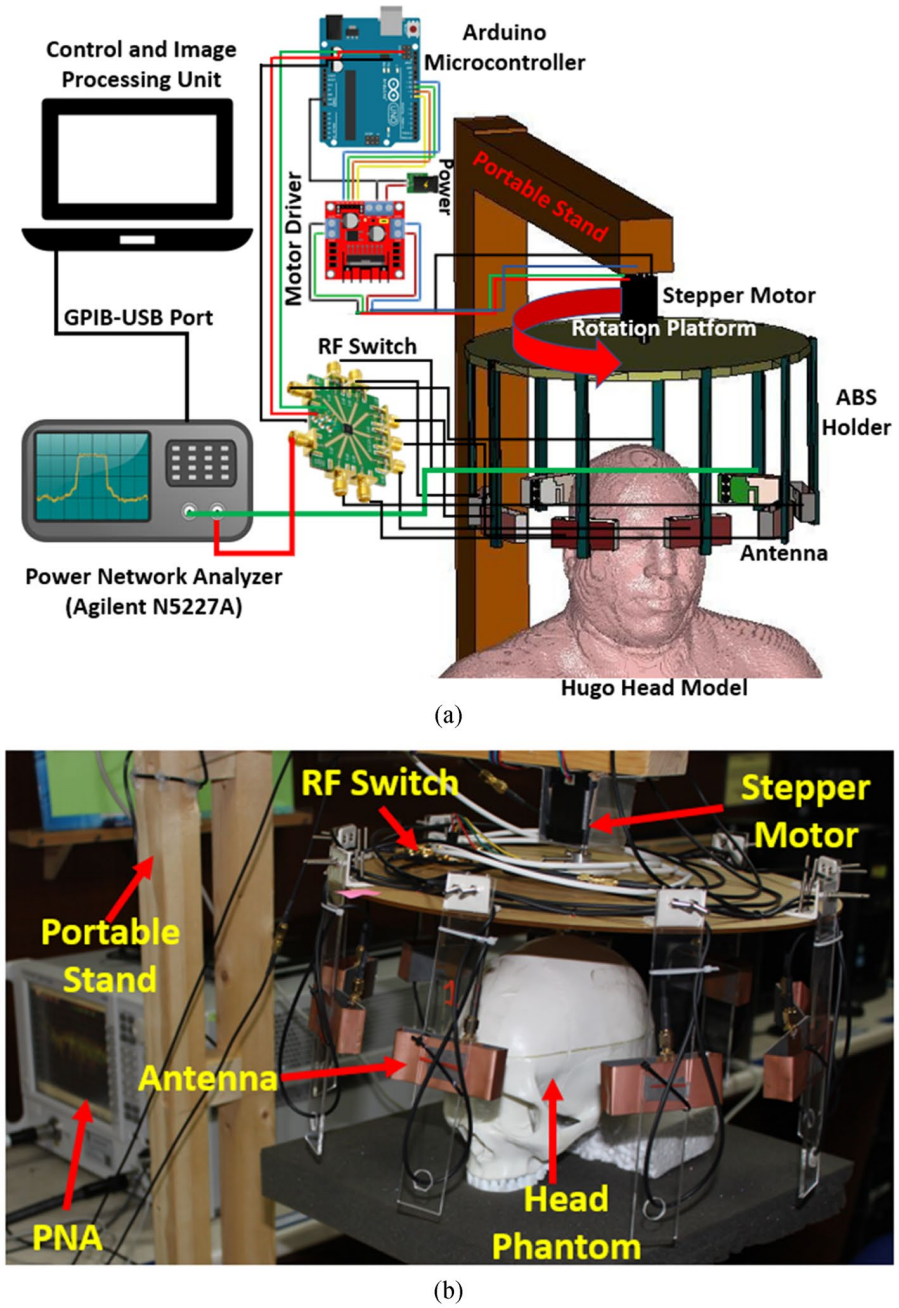
The proposed portable head imaging system (**a**) simulation model, and (**b**) perspective view with human head phantom.

In the experimental analysis, a stepper-motor based mounting stand, an SP8T RF switching system, and a personal computer-based image processing unit are also combined with the system. Nine transparent plastic sticks with antennas are installed on the rotating platform. The head phantom is placed in the middle with a stand where the mechanical rotation platform rotates in polar coordinates from 0 to 2π around it, depicted in Fig. 23 (simulation and experimental model). The data (S21, S31, S41, S51, S61, S71, and S81) are collected at each 7.2°, and 50 equal points, covering the total 360°. The Agilent E8358A Power network analyzer (PNA) works as a transceiver that generates EM signals through the transmitting antenna. This PNA is also connected to the personal computer via GPIB port that processes the received data for image reconstruction. The IC-CF-DMAS algorithm is analyzed based on microwave signal contrast where the comparisons will take place between the reference microwave signal using numerical simulation of full-wave time-domain and the scattered signal from the computational phantom. A successive approximation method will be obtained in order to get the exact position and size of the damaged tissue.

In this study, the radial distance and height from the center of the bottom surface of the imaging domain are optimized for a given setup of circular antenna arrays. This can be interpreted as adjusting the focusing of a camera with respect to different lenses, which are analogous to the antennas. Two sets of data are recorded for the empty setup for the calibration process. The difference between the two data sets is calculated as a measure of noise present in the imaging system. The measured noise is processed using the conventional Coherence Factor Delay and Sum (CFDAS) to reconstruct a scattering intensity map of the imaging domain. Since the data is derived from a blank setup, meaning there are no scattering objects in the imaging domain, the image derived from the difference should yield a blank output. Any recorded scattering intensity must be considered clutter has to be minimized to remove any multipath flares or false positives due to background noise. Thus, the fitness function for the optimization algorithm is the average scattering intensity in the reconstructed image from the signal difference. The radius of the observation array is optimized along the radial length of the antenna. The radius of the circular disc (Antenna mounting sphere), upon which the entire array is placed, is considered the upper limit of the radial dimension during optimization, and the lower limit is considered the difference between disc radius and antenna length. The height dimension of the optimization is considered from the feed point of the antennas and measured along with the height of the array housing cylinder. The algorithm parameters will be working according to the key criteria for compatibility for the phantom imaging. The sample scattering parameters, represented as *S (f, rx, φ)* will be divided into two matrices, where they are depending on the *S*_*odd*_* (f, rx, φ*_*odd*_*)* and *S*_*even*_* (f, rx, φ*_*even*_*)*, individually, where *φ*_*odd*_ = 1,3,5,…N_*φ*_-1, and *φ*_*even*_ = 2,4,6,…N_*φ*_. Therefore, the *S*_*even*_ is considered as offset illumination and *S*_*odd*_ is considered as initial illumination. The Inverse Fourier Transform method is then applied to the signal to convert the frequency domain into time domain mode by generating *Γ(t, rx, φ*_*odd*_*)*. Later, *Γ(t, rx, φ*_*odd*_*)* is be analyzed and process with the proposed algorithm for image reconstruction. The proper delay is generated by dividing the total distance *l*, with the background medium air and the dielectric constant is *ε*_*r*_.

19 $$\tau (i,r_{x} ,\varphi_{odd} ) = \frac{{\sqrt {\varepsilon_{r} } (p_{{C - Rx\varphi_{odd} }} (i,r_{x} ,l))}}{c}$$

Here, *c* is the speed of light.

The delay is calculated from the anticipated smallest distance and the reflected signal from *C(i).* The delays are further added to the signals for delivering the proper delayed signal. After multiplying the paired delayed signal, they are summed for determining the scattering intensity at the allotted point in the region of interest, as shown in the following equation.

20 $$\Upsilon_{DMAS} (i) = \int\limits_{ - \infty }^{\infty } {\sum\limits_{{\varphi_{odd} = 1}} {\sum\limits_{rx = 1} {\left[ {\Gamma \left( {t - \frac{{\tau (i,r_{x} ,\varphi_{odd} )}}{\Delta t},r_{x} ,\varphi_{odd} } \right)} \right]dt} } }$$

The below equation calculates the coherence factor (CF), which is modelled to more CF channels with more weights at each individual stage within the imaging domain.:

21 $$CF(i) = \frac{{\Upsilon_{DMAS} (i)}}{{\int\limits_{ - \infty }^{\infty } {\sum {_{{\varphi_{odd} }} \sum {_{rx} \Gamma \left( {t - \frac{{\tau (i,rx,\varphi_{odd} )}}{\Delta t},rx,\varphi_{odd} } \right)} } } dt}}$$

From then on, the scattering intensity map will be computed by the next formula:

22 $$\Upsilon_{CF - DMAS} (i) = CF(i) \cdot \Upsilon_{DMAS} (i)$$

The distance inverse weighting will be utilized for the reflection of the 3D Green function for electromagnetic waves.

23 $$\Upsilon^{\prime } (i) = \int\limits_{C} {\frac{{\Upsilon_{CF - DMAS}^{n - 1} (i)}}{{1 + p_{C - C} (i,j)}}dj}$$

After that, the modified delay will be estimated by the subsequent formula:

24 $$\tau^{\prime } (i,rx,\varphi_{odd} ) = \tau (i,rx,\varphi_{odd} ) + \frac{{\Upsilon^{\prime } (i)}}{c}$$

The Coherence Factor will be computed, and the scattering intensity map will be assessed as below:

$$\Upsilon_{CF - DMAS}^{n} (i) = CF(i) \cdot \Upsilon_{DMAS}^{n} (i)$$

In accordance with the modified delays, the scattering strength map will be reconstructed.

Lastly, the closure requirements test for convergence. Equations (23)-(25) are assessed for n = 1 iteratively, 2…. 7.

25 $$E_{\Upsilon } = \sum\nolimits_{\forall i} {\left| {\Upsilon_{CF - DMAS}^{n} } \right. - \left. {\Upsilon_{CF - DMAS}^{n - 1} } \right|}$$

Furthermore, it is crucial to consider the matching medium for the proposed EM imaging system. The noise and distance between antenna radiating elements and head phantom are considered and optimized in this study.

Therefore, the Additive White Gaussian Noise (AWGN) channel is imagined as the background medium due to the Power Spectral Density (PSD) and proper delay of IC-CF-DMAS algorithm. For the proposed EM imaging system, the noise insertion or noise source possibilities are hypothetically assumed and divided into three categories, such as, electrical, vibration and shot noise. The noise PSD is calculated as the square root of the noise power where it could be left or right sided PSD. However, the AWGN channel considers both-sided PSD and therefore, a general mathematical model is represented and applied to the timing error by considering an ideal environment. For a general AWGN channel, $P_{w} = N_{0} \cdot \frac{{F_{s} }}{2}$ where *P*_w_ represents the noise power, *N*_0_ depends on the noise power over bandwidth, and Fs represents sample bandlimited signal frequency. Finally, for the proposed EM imaging system, the following Eq. (26) is updated based on the stated situation. At this point, the AWGN channel power is multiplied with the matching medium dielectric constant.

26 $$\tau (i,tx,rx,\varphi_{odd} ) = \frac{{\sqrt {E_{AWGN} } P_{w} (P_{{Tx\varphi_{odd} }} - {}_{c}(i,tx,l) + p_{{c - Rx\varphi_{odd} }} (i,rx,l))}}{c}$$

**Figure 24:**
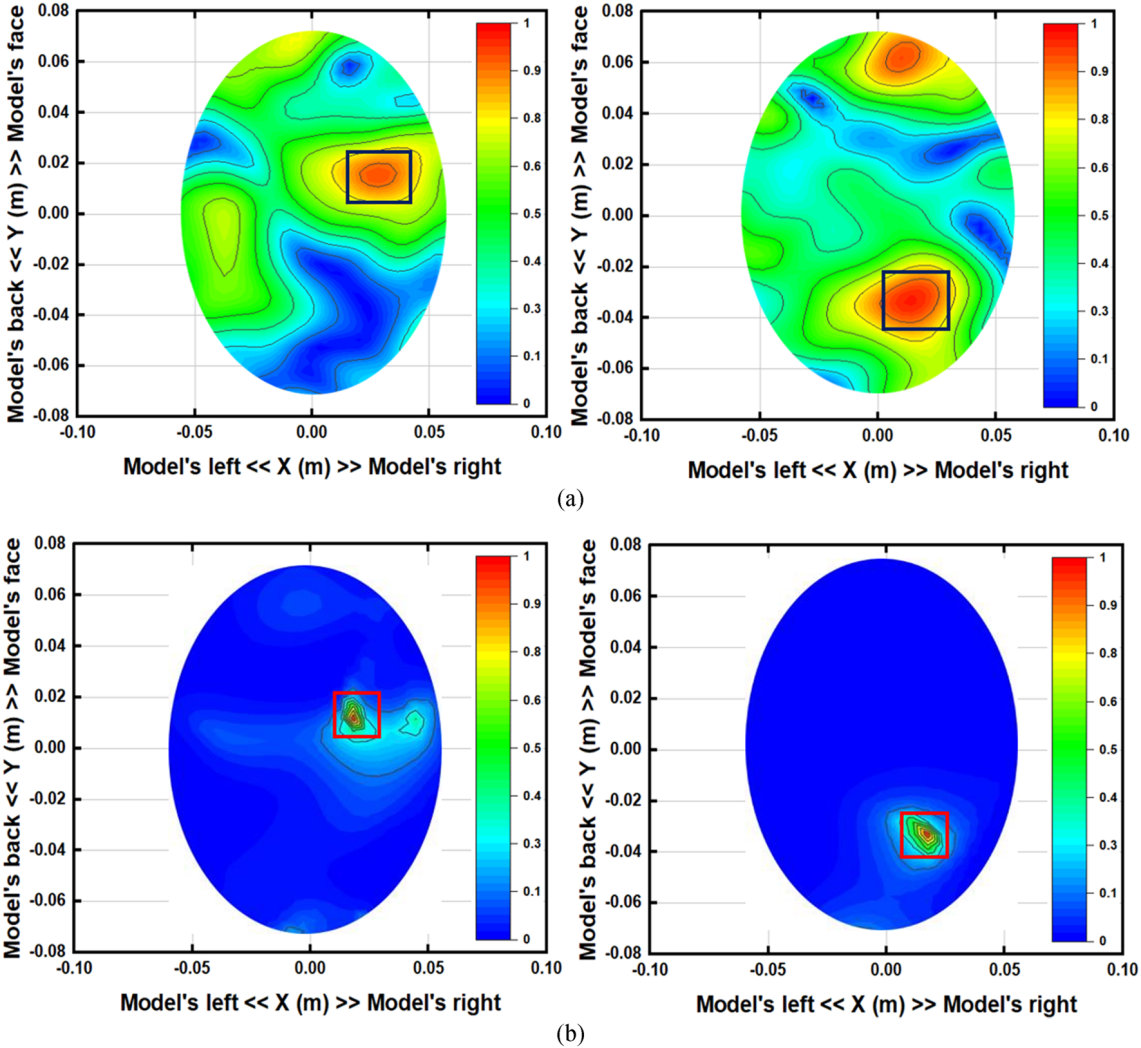
The reconstructed images using the proposed antenna in two different positions. (**a**) Applying conventional DMAS and (**b**) applying IC-CF-DMAS. The small red square marks the inserted hemorrhage.

By differentiating the collected backscattered signals from the healthy and unhealthy phantom, the unhealthy tissue is identified as hemorrhage, which can clearly be detected from this imaging analysis. Figure 24 represents the reconstructed images from two different head phantoms. Two different locations are used for the haemorrhages. Conventional DMAS and IC-CF-DMAS have been applied to validate the detection performance. The obtained reconstructed images verify that the proposed portable microwave head imaging system can detect and locate the position of the haemorrhages inside the head model.

The performance comparison among the proposed and other antennas in microwave head imaging is presented in Table 3.

**Table 3:** Comparison among proposed and other existing works in microwave head imaging system.

Ref	Structure	Dim	Substrate	Substrate layer	MTM inclusion	Fractional Bandwidth (%)	Realized gain (avg.) dBi	FF (%)	Max. SAR(W/kg)	Head phantom
^66^	Tapered Slot	95 × 90 × 1.28 mm^3^	Rogers RO3010	1	No	120%	–	–	–	Tissu-mimicking
^67^	3D	70 × 30 × 15 mm^3^	GIL 1023	2	No	102.2%	< 4	–		Tissu-mimicking
^22^	3D	80 × 20 × 10 mm^3^	FR4	2	No	67%	3.15	80		Tissu-mimicking
^44^	3D	70 × 15 × 15 mm^3^	Rogers RT3000	2	No	97%	3	–		Tissu-mimicking
^45^	3D	25 × 25 × 10.5 mm^3^	Air	1	No	9.35%	6.6	–		Tissu-mimicking
^68^	Microstrip	29.99 × 29.99 × 0.59 mm^3^	FR4	1	No	2.84%	< 3	–	0.332	No
^23^	3D	68 × 68 × 22.5 mm^3^	FR4	5	No	51.85%	–	–	0.0147	Inhomogeneous
^32^	Microstrip	50 × 44 × 1.524 mm^3^	Rogers RO4350B	1	No	74.3%	< 4	98	0.233	No
^29^	Microstrip	79 × 68.28 mm^3^	FR4	1	No	10%	–	–	–	Heterogeneous
^30^	Brick	50 × 70 × 50 mm^3^	FR4	1	No	40%	–	–	–	Homogeneous
This Work	3D	70 × 30 × 15 mm^3^	Rogers RT5880	1	Yes	80%	> 4	83	0.071	Tissue-mimicking

## Conclusion

A portable microwave head imaging system with MTM loaded compact 3D antenna is presented. The main components of the system are array of nine antennas, tissue-mimicking head phantom, a power network analyzer, and a computing tool to collect and store the scattered data for post-processing. The developed antenna has symmetrical top and ground elements with a 1 × 3 finite array MTM unit cell structure that increases the antenna bandwidth, realized gain, and efficiency. The operating bandwidth for the measured antenna is from 1.95 to 4.5 GHz with a directional radiation pattern. The tissue-mimicking head phantom is utilized to validate the antenna performance when placed in proximity. A set of scattered data is collected from the different antenna positions that are later post-processed with the IC-CF-DMAS beamforming algorithm to reconstruct the hemorrhage images. The hemorrhage detection from the reconstructed images shows the feasibility of the proposed microwave head imaging system as a portable platform.
